# The neutralizing breadth of antibodies targeting diverse conserved epitopes between SARS-CoV and SARS-CoV-2

**DOI:** 10.1073/pnas.2204256119

**Published:** 2022-08-16

**Authors:** Hualong Xiong, Hui Sun, Siling Wang, Lunzhi Yuan, Liqin Liu, Yuhe Zhu, Jinlei Zhang, Yang Huang, Ruoyao Qi, Yao Jiang, Jian Ma, Ming Zhou, Yue Ma, Rao Fu, Siping Yan, Mingxi Yue, Yangtao Wu, Min Wei, Yizhen Wang, Tingting Li, Yingbin Wang, Zizheng Zheng, Hai Yu, Tong Cheng, Shaowei Li, Quan Yuan, Jun Zhang, Yi Guan, Qingbing Zheng, Tianying Zhang, Ningshao Xia

**Affiliations:** ^a^State Key Laboratory of Molecular Vaccinology and Molecular Diagnostics, National Institute of Diagnostics and Vaccine Development in Infectious Diseases, School of Public Health, School of Life Sciences, Xiamen University, Xiamen 361102, China;; ^b^Guangdong-Hongkong Joint Laboratory of Emerging Infectious Diseases/Joint Laboratory for International Collaboration in Virology and Emerging Infectious Diseases, Joint Institute of Virology (Shantou University and University of Hong Kong), Shantou University, Shantou 515063, China;; ^c^State Key Laboratory of Emerging Infectious Diseases, University of Hong Kong, Hong Kong 999077, China;; ^d^Research Unit of Frontier Technology of Structural Vaccinology, Chinese Academy of Medical Sciences, Xiamen 361102, China

**Keywords:** SARS-CoV-2, SARS-CoV, broad neutralizing antibody, structure, conversed epitopes

## Abstract

Antibody therapeutics for the treatment of COVID-19 have been highly successful. However, the recent emergence of the Omicron variant has posed a challenge, as it evades detection by most existing SARS-CoV-2 neutralizing antibodies (nAbs). Here, we successfully generated a panel of SARS-CoV-2/SARS-CoV cross-neutralizing antibodies by sequential immunization of the two pseudoviruses. Of the potential candidates, we found that nAbs X01, X10, and X17 offer broad neutralizing potential against most variants of concern, with X17 further identified as a Class 5 nAb with undiminished neutralization against the Omicron variant. Cryo-electron microscopy structures of the three antibodies together in complex with each of the spike proteins of the prototypical SARS-CoV, SARS-CoV-2, and Delta and Omicron variants of SARS-CoV-2 defined three nonoverlapping conserved epitopes on the receptor-binding domain. The triple-antibody mixture exhibited enhanced resistance to viral evasion and effective protection against infection of the Beta variant in hamsters. Our findings will aid the development of antibody therapeutics and broad vaccines against SARS-CoV-2 and its emerging variants.

## Significance

This study developed a rational antibody cocktail containing three cross-neutralizing antibodies that can bind to the receptor-binding domain of SARS-CoV and SARS-CoV-2. Cryo-electron microscopy structures of the triple-antibody cocktail in complex with the spike proteins of prototyped SARS-CoV-2, Delta, Omicron, and SARS-CoV defined three nonoverlapping conserved neutralizing epitopes. This knowledge will facilitate the development of broad-acting antibody therapeutics and vaccines against SARS-CoV-2 and emerging variants.

By June 2022, the COVID-19 pandemic, caused by SARS-CoV-2, had resulted in more than 6 million deaths worldwide (1–3). Monoclonal antibodies (mAbs) isolated from SARS-CoV-2–infected individuals were effective as both therapeutic and prophylactic agents against SARS-CoV-2 (4–6), with several neutralizing antibodies (nAbs), including sotrovimab (7) and bamlanivimab (8), and nAb mixtures, including casirivimab–imdevimab (9) and bamlanivimab–etesevimab (10), approved under Emergency Use Authorization (EUA) for the treatment of patients with COVID-19. However, the constant evolution and genetic drift of SARS-CoV-2 has resulted in the emergence of many variants of concern (VOCs) depending on the main protein of the SARS-CoV-2 prototype strain, including the Alpha (B.1.1.7), Beta (B.1.351), Gamma (B.1.1.28), Delta (B.1.617.2), and Omicron (B.1.1.529) variants, the latter of which has become the major concern. Indeed, the Omicron variant harbors numerous residue substitutions in the spike (S) protein, with at least 15 mutations highly intertwined with common neutralizing epitopes in the receptor-binding domain (RBD) (11, 12). Various studies have reported that critical mutations within these VOCs prohibit the potent mAb neutralization that works against the ancestral isolate, leading to a much-diminished protective efficacy of antibody therapeutics against SARS-CoV-2 (13–20). Therefore, there is still a pressing need for nAbs with broader neutralizing breadth against current VOCs and future emerging variants.

The trimeric S protein mediates SARS-CoV-2 entry into host cells via the RBD, which binds to the angiotensin-converting enzyme 2 (ACE2) receptor (1, 21, 22). Given its role, the RBD is regarded as a critical target for the development of therapeutics and vaccines against COVID-19. Indeed, numerous potently neutralizing mAbs are shown to target the receptor-binding motif (RBM) on the RBD, thereby efficiently inhibiting the S protein from binding to ACE2 to minimize or prohibit infection (4, 23, 24). However, VOCs frequently possess mutations within the RBM, which significantly reduces the neutralization breadth of mAbs that recognize this site (13–18, 25). Nevertheless, of the five classes of RBD-targeting nAbs (4, 26), three classes—represented by S309 (27), S2X259 (28), and S2H97 (26)—offer cross-neutralization against SARS-CoV-2 and SARS-CoV and thus can also inhibit infection from most VOCs. Consequently, it is assumed that epitopes within these sites are highly conserved among *Sarbecoviruses* and that antibody mixtures comprising representative nAbs that bind to these conserved epitopes may be able to prevent SARS-CoV-2 variants and other zoonotic “spillover” SARS-like viruses. In addition, under the selective pressure of antibody therapeutics, such as screening, the emergence of avoidance mutations becomes an important issue that should be considered. Such antibody avoidance studies in vitro have strongly supported the rationale of antibody mixtures consisting of noncompeting antibodies to avoid the development of resistance (13, 15, 29).

nAbs reported to date have been primarily obtained from the human humoral immune response induced by vaccination or natural infection of SARS-CoV or SARS-CoV-2. The singular exposure of *Sarbecoviruses* at a time has hindered the generation of cross-neutralizing mAbs (26–28). Based on influenza virus research (30–32), the development of cross-neutralizing antibodies may benefit from the combined immunization of SARS-CoV and SARS-CoV-2 in sequence, offering insight into immune-focusing on conserved epitopes between the two virus strains.

In this study, we focus on the conserved epitopes between SARS-CoV-2 and SARS-CoV. To this end, we generated a panel of broad-neutralizing antibodies (bnAbs) against SARS-CoV, SARS-CoV-2, and VOCs from sequentially immunized mice. Three representative bnAbs, X01, X10, and X17, were further identified to offer potent cross-neutralizing activity against most VOCs but with a decreased neutralization breadth against Omicron. High-resolution cryo-electron microscopy (cryo-EM) structures revealed three nonoverlapping conserved epitopes and defined the structural basis for the neutralization breadth of the three bnAbs. Using these three bnAbs in a mixture efficiently resisted viral escape and protected Syrian hamsters against challenge with the SARS-CoV-2 Beta variant. Thus, by taking advantage of conserved epitopes, our results expand upon the current therapeutic strategy and offer a way to cope with circulating and future emerging SARS-CoV-2 VOCs as well as any potential spillover zoonotic SARS-like viruses. This study thus highlights the potential utility of diverse, conserved epitopes for effective vaccine design.

## Results

### Sequential Immunization of SARS-CoV and SARS-CoV-2 Elicits Cross-Neutralizing Antibodies.

SARS-CoV-2 and SARS-CoV cross-neutralizing mAbs against the RBD were generated by carrying the SARS-CoV and SARS-CoV-2 S proteins on the recombinant vesicular stomatitis virus (VSV) pseudovirus, referred to as rVSV-SARS and rVSV-SARS2, respectively, as previously reported (33). Mice were alternately immunized with each of the two purified pseudoviruses at 1-wk intervals, using rVSV-SARS as the priming immunogen (*SI Appendix*, Fig. S1). After three doses of both rVSV-SARS and rVSV-SARS2, the sequentially immunized mice were killed to separate spleen cells. For the selection of SARS-CoV/SARS-CoV-2 cross-neutralizing mAbs, hybridoma cell pools were built by cell fusion, and we finally obtained a total of 34 cross-neutralizing mAbs from the total of 100 hypridoma cell clones for further evaluation.

**Fig. 1.**
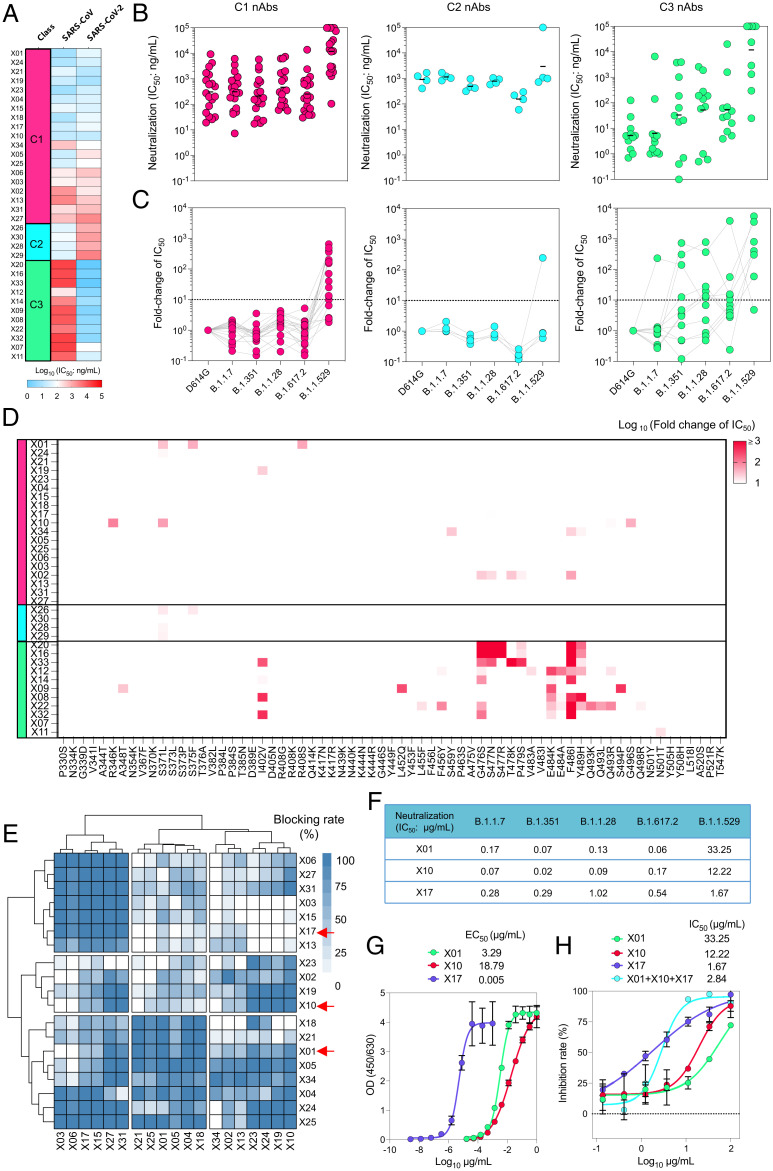
Characterization of bnAbs induced by sequential immunization of pseudoviruses of SARS-CoV and SARS-CoV-2. (*A*) Classification of a panel of 34 nAbs based on their cross-neutralization of SARS-CoV and SARS-CoV-2. The neutralization potencies of nAbs were evaluated by rVSV-based pseudoviruses of SARS-CoV and SARS-CoV-2. The IC_50_ values ranging from 1 ng/mL to 50 μg/mL are represented in blue to red, respectively. nAbs were classified into classes (C1, C2, and C3), based on the fold change of IC_50_ values against SARS-CoV-2 related to SARS-CoV (C1: 0.1–10; C2: <0.1; and C3: >10). (*B* and *C*) IC_50_ values of three classes of nAbs against pseudoviruses of the D614G strain and VOCs B.1.1.7, B.1.351, B.1.1.28, B.1.617.2, and B.1.1.529 (*B*) and IC_50_ fold change compared with that of the prototyped SARS-CoV-2 (*C*). nAbs of C1, C2, and C3 are colored in magenta, cyan, and green, respectively. Black lines in (*B*) indicate the geometric means and black dashed lines in (*C*) indicate a fold-change of 10 ×. (*D*) Interference of single-point mutations on neutralization potencies of nAbs in C1, C2, and C3. IC_50_ values for different nAbs were determined against VSV pseudoviruses carrying SARS-CoV-2 S protein with single-residue substitutions. Fold change in IC_50_ values of mutant pseudoviruses (related to D614G control) were calculated. The abscissa shows different mutant residues. (*E*) Cross-blocking matrix for C1 nAbs. The concentrations of blocking nAbs (row) and detection nAbs (column) were 500 μg/mL and 10 ng/mL, respectively. The intensity of cyan indicates blocking strength ranging from 0% (no blocking, white) to 100% (complete blocking, dark cyan). nAbs were classified into three major clusters, using R (version 3.6.3) by the ward.D2 method. Red arrows indicate representative nAbs (X01, X10, and X17). (*F*) Neutralization potencies of X01, X10, and X17 against SARS-CoV-2 VOCs, including B.1.1.7, B.1.1.28, B.1.351, B.1.617.2, and B.1.1.529. (*G* and *H*) Binding activities (*G*) and neutralization potencies (*H*) of X01, X10, and X17 against the SARS-CoV-2 Omicron variant. The EC_50_ and IC_50_ values were calculated by Prism software using nonlinear regression (four parameters). The experiments in (*A*, *B*, *G*, and *H*) were repeated in duplicate.

Given the diverse neutralization potential against SARS-CoV and SARS-CoV-2, the 34 cross-neutralizing mAbs were categorized into three classes: class 1 (C1), C2, and C3 (Fig. 1*A* and *SI Appendix*, Table S1). Most mAbs (19 of 34) were classified into C1, which showed comparable neutralizing efficacies against SARS-CoV-2 and SARS-CoV, with half-maximal inhibitory concentration (IC_50_) values differing within one order of magnitude; these cross-neutralizing mAbs thus recognized highly conserved epitopes found within SARS-CoV-2 and SARS-CoV. Conversely, C2 and C3 contained weakly cross-neutralizing mAbs and showed biased neutralization potencies against SARS-CoV (C2) or SARS-CoV-2 (C3) (Fig. 1 *B* and *C* and *SI Appendix*, Table S1). We next determined the broad neutralizing potencies of these mAbs against pseudoviruses of VOCs including B.1.1.7 (Alpha), B.1.351 (Beta), B.1.1.28 (Gamma), B.1.617.2 (Delta), and B.1.1.529 (Omicron). Notably, C1 cross-neutralizing mAbs showed comparable neutralizing efficacies against pseudoviruses of VOCs except the Omicron variant when compared with the D614G strain (Fig. 1*B*, *Left*), with IC_50_ values differing within one order of magnitude (Fig. 1*C*, *Left*). These results indicated that C1 nAbs not only ensured strong neutralization activities but also effectively prevented the escape of SARS-CoV-2 variants. Notably, all C2 mAbs showed moderate neutralizing potencies against SARS-CoV-2 and VOCs, with IC_50_ values greater than 100 ng/mL (Fig. 1 *B* and *C*, *Middle*). The SARS-CoV-2–biased C3 nAbs were less resistive to viral escape than C1 nAbs, with decreased neutralizing potency against VOCs as compared with the wild-type (WT) D614G strain (Fig. 1 *B* and *C*, *Right*).

From these findings, we can infer that C1 nAbs are more resistant to the variety of mutations found in VOCs as well as other variants of interest (VOIs). To confirm this, we sought to investigate single mutations on the RBD and their influence on mAb neutralization. A total of 67 RBD single-point mutations observed with high frequency since the COVID-19 outbreak were included in this study (34). A set of 67 corresponding mutant SARS-CoV-2 pseudoviruses carrying these single-point mutations were constructed and used to evaluate the efficacy of all three classes of nAbs. Overall, the neutralization results were similar to those assessed against VOCs. Of the three classes of nAbs, C1 and C2 nAbs were resistant to the majority of mutations and were therefore identified as broad neutralizers. C3 nAbs, however, showing a biased neutralization to SARS-CoV-2, demonstrated much lower neutralization potencies against the mutations to varying degrees (Fig. 1*D*). Of interest, the antigenic site spanning residues 470 to 490 (site 470–490) was determined as a “hit area” for C3 nAbs, because single mutations within this site caused a significant decrease in neutralizing potency (Fig. 1*D* and *SI Appendix*, Fig. S2). Specifically, the sensitivity of C3 nAbs to mutations to the E484 residue might be responsible for the diminished neutralization against the Beta and Gamma variants. In addition, we noted that the neutralization efficacy of seven of the 11 C3 nAbs was abolished in variants bearing an F486I mutation in the SARS-CoV-2 RBD. This mutation was a reversal of the isoleucine-to-phenylalanine substitution at this position found between SARS-CoV and SARS-CoV-2 (Fig. 1*D* and *SI Appendix*, Fig. S2). Thus, these results suggest that residue 486 plays a critical role in mediating the antigenic discrepancy noted between SARS-CoV-2 and SARS-CoV. Taken together, the sequential immunization with SARS-CoV and SARS-CoV-2 successfully elicits cross-neutralizing antibodies that are resistant to all VOCs that emerged prior to Omicron. In addition, the epitopes recognized by C1 nAbs should be explored in further detail for their therapeutic and clinical efficacy.

### Cross-Neutralizing Antibodies Belong to Three Clusters with Different Resistance to the Omicron Variant.

Given the potent broad neutralization efficacy of C1 mAbs, we next sought to characterize their function in detail. To this end, an RBD-based competitive binding assay was carried out using the C1 cross-neutralizing antibodies further subdivided into three major clusters (clusters 1, 2, and 3) (Fig. 1*E*). These three clusters of nAbs showed varied extents of epitope overlapping. Asymmetric competition (one mAb blocks another from binding, but the latter does not block the former) was also observed, presumably due to affinity differences between nAbs. In consideration of the unbiased neutralizing potency against SARS-CoV and SARS-CoV-2, X17, X10, and X01 were selected as representative nAbs for clusters 1, 2, and 3, respectively. Of the three clusters, representative nAb X17 (cluster 1) poorly blocked the binding of receptor ACE2 onto the S protein, whereas X10 and X01 (clusters 2 and 3, respectively), efficiently blocked the binding of ACE2 (*SI Appendix*, Fig. S3). Altogether, we surmised that the three clusters recognize three nonoverlapping sites on the RBD, with there being some potential overlap of the ACE2 binding epitopes for X01 (cluster 3) and X10 (cluster 2). In addition, all three representative nAbs were determined as being potent cross-neutralizing antibodies against both SARS-CoV and SARS-CoV-2, with IC_50_ values less than 0.1 μg/mL (*SI Appendix*, Table S1). Notably, X01 showed excellent neutralization, probably due to its effective blocking potency against ACE2 binding (half-maximal effective concentration [EC_50_] of 0.17 μg/mL) (*SI Appendix*, Fig. S3).

We then confirmed the broadly neutralizing efficacies of these three representative nAbs against SARS-CoV-2 VOCs, including the Omicron variant. X01 and X10 showed potent neutralizing activities, with IC_50_ values ranging from 0.02 to 0.17 μg/mL against VOCs (B.1.1.7, B.1.351, B.1.1.28, and B.1.617.2), and these values were significantly higher than those for X17 (IC_50_ ranging from 0.28 to 1.02 μg/mL) (Fig. 1*F*). Synchronously, we noted that few point mutations could destroy their neutralization activities, which further demonstrated the broad neutralizing activities of these three nAbs (Fig. 1*D*).

The currently dominant VOC, Omicron, which contains an unprecedented 15 mutations in the RBD, is highly resistant to neutralization by plasma from vaccinated individuals, convalescent sera, and most reported nAbs (11, 12). Unfortunately, X01 and X10 showed only weak binding activities against the Omicron S protein, with EC_50_ concentrations of 3.29 and 18.79 μg/mL, respectively. In contrast, X17 showed a strong interaction with the Omicron S protein, with an EC_50_ value of 0.005 μg/mL (Fig. 1*G*). X17 showed a comparably lower neutralizing efficacy (IC_50_ 1.67 μg/mL) than the WT and other VOCs, while neutralization almost completely lost for X01 and X10 against the Omicron variant (with IC_50_ of 33.25 and 12.22 μg/mL, respectively) (Fig. 1*H*). Overall, the antigenic mutations of the Omicron variant hampered the neutralizing breadths of nAbs X01 and X10 but not X17.

### Cross-Neutralizing Antibodies Define Three Nonoverlapping Conserved Epitopes on RBD.

**Fig. 2.**
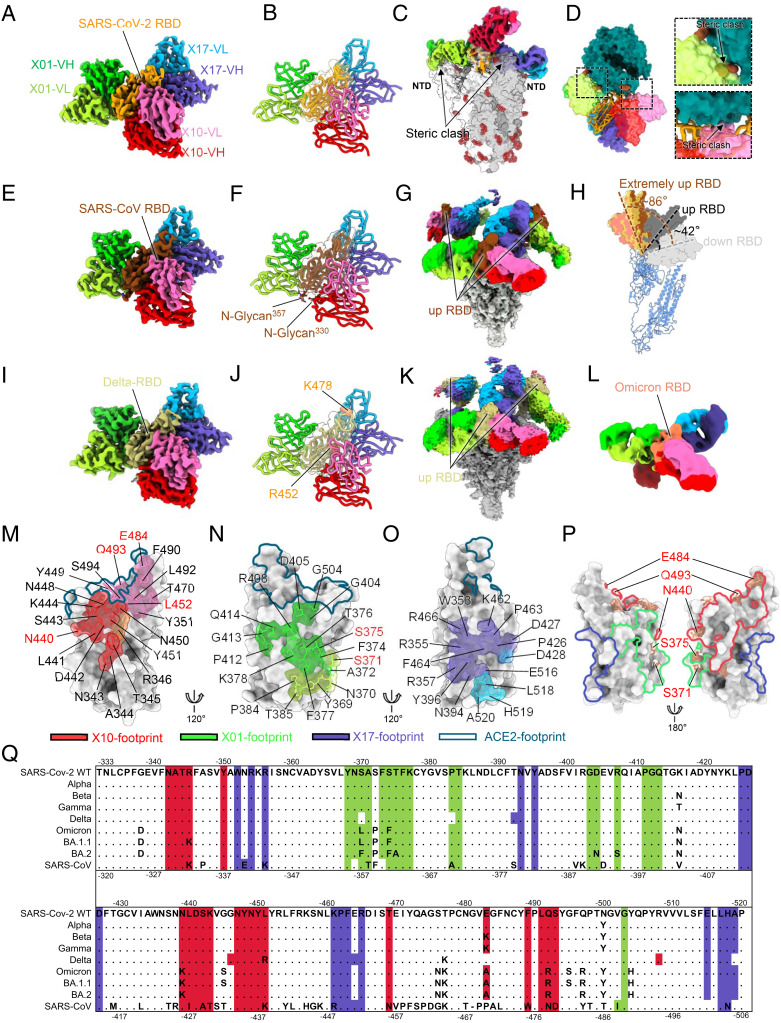
Cryo-EM structures of the three-antibody combination in complex with the S proteins of prototyped SARS-CoV-2 and SARS-CoV as well as SARS-CoV-2 Delta and Omicron variants. (*A* and *B*) Domain-colored cryo-EM map (*A*) and cartoon representation (*B*) of the cryo-EM structure of SARS-CoV-2-S:X10:X01:X17. The surface representation of RBD is transparent. The S protein was resolved in monomeric form. (*C*) Superimposition of the SARS-CoV-2 spike (PDB accession code 6VSB) onto the density map of SARS-CoV-2-S:X10:X01:X17 indicates steric clashing between Fab X01 and X17 and the neighboring N-terminal domain (NTD). (*D*) Superimposition of structures of the ACE2:RBD (PDB: 6M0J) and SARS-CoV-2-S:X10:X01:X17 depicts steric clashing between Fab X01 and Fab X10 with ACE2. (*E–G*) Cryo-EM structure of SARS-CoV-S:X10:X01:X17. Local refinement density map of the interface (*E*), the original global refinement density map (*G*), and a cartoon representation of the model (*F*) are shown. (*H*) Binding of three nAbs to the trimeric S proteins caused all three RBDs to exhibit an extremely opened “up” orientation of ∼90° (relative to the closed RBD), as compared with ∼42° for the canonical “up” RBD. (*I*–*K*) Cryo-EM structure of Delta-S:X10:X01:X17. Local refinement density map of the interface (*I*), the original global refinement density map (*K*), and a cartoon representation (*J*) of the model are shown. (*L*) Domain-colored cryo-EM map of the cryo-EM structure of Omicron-S:X10:X01:X17. (*M*–*O*) Footprints of X10 (*M*), X01 (*N*), and X17 (*O*) on SARS-CoV-2 WT-RBD. The RBD is presented in surface representation (gray). Residues involved in the nAb interactions are shown in stick representation with a transparent surface. Contact regions of the heavy chains, light chains, and both chains of X10 on the RBD are colored in red, pink, and brown, respectively. The contact regions of the heavy and light chains of X01 are colored in lime and yellow-green, respectively. The contact regions of the heavy and light chains of X17 are colored in slate blue and sky blue, respectively. The ACE2-binding site (base on PDB accession code 7C8D) is indicated by a black dotted line. (*P*) There is no overlap in the footprints of X10 (red line), X01 (green line), and X17 (slate blue line) on the WT-RBD (gray surface representation). Mutation sites of the Omicron variant on the RBD are highlighted in coral. (*Q*) Sequence alignment of the RBDs of SARS-CoV-2, VOCs, and SARS-CoV. Strictly conserved residues are shown as dots, and epitopes of the three nAbs are highlighted using the color scheme in (*P*).

To define the conserved epitopes of the three cross-neutralizing antibodies, we first employed a cryo-EM approach to ascertain the complex structures of three-nAb combination in binding to the WT S proteins of SARS-CoV-2 (SARS-CoV-2-S) and SARS-CoV (SARS-CoV-S). Cryo-EM structures of SARS-CoV-2-S and SARS-CoV-S in complex with three nAbs simultaneously were obtained at resolutions of 3.48 Å (Fig. 2 *A*–*D* and *SI Appendix*, Figs. S4 and S8 and Table S2) and 3.83 Å (Fig. 2 *E*–*G* and *SI Appendix*, Figs. S5 and S8 and Table S2), respectively. Of interest, the simultaneous binding of all three nAbs to SARS-CoV-2-S caused a dissociation of the trimeric S protein, which allowed us to solve the structure of the monomeric S protein in complex with the three nAbs (SARS-CoV-2-S:X01:X10:X17) (*SI Appendix*, Fig. S4). Superimposition of the atomic model of SARS-CoV-2-S:X01:X10:X17 onto the structure of the trimeric S protein showed conspicuous antibody-induced steric clashes mediated by both X10 and X17 but not X01, suggesting that X10 and X17 may harbor a dissociation potency toward the S trimer (Fig. 2*C*). Furthermore, we saw that X01 and X10, but not X17, occupied the space required for ACE2 binding (Fig. 2*D*), thereby blocking ACE2 action (*SI Appendix*, Fig. S3).

As for the SARS-CoV-S:X01:X10:X17 reconstruction, we could classify both trimeric S (∼49%) and dissociated monomer S (∼6%) proteins in complex with the three nAbs (*SI Appendix*, Fig. S5), suggestive of a less-potent dissociation efficacy of the nAbs on SARS-CoV-S as compared with SARS-CoV-2-S.

Next, particles of the trimeric S complexes were selected for further reconstruction (*SI Appendix*, Fig. S5). In the 3.74 Å structure of the trimeric SARS-CoV-S:X10:X01:X17, all three RBDs of the S proteins in the trimer were noted to be in the “up” conformation and bound by three antigen-binding fragments (Fabs) simultaneously; only one RBD was found with strong Fabs densities, while the other two RBDs showed relatively weaker densities of bound Fabs (Fig. 2*G* and *SI Appendix*, Fig. S9*B*). We speculated that only one RBD on the S protein was saturated by Fabs, and this was further refined to push the local resolution (Fig. 2*E* and *SI Appendix*, Fig. S5). Notably, three RBDs on the nAb-bound trimeric SARS-CoV-S were raised to extremely open states (compared to a “closed” RBD conformation), ranging from 85° to 87°; this was compared with 42° for a typical open RBD in an S protein (Fig. 2*H*). Together, these findings suggest that only sufficiently opened RBDs can accommodate all three Fabs—especially for X10 and X17—simultaneously and avoid steric clashing and trimeric S disruption.

We next investigated the simultaneous binding potential of three nAbs to the S proteins of the Delta (Delta-S) and Omicron (Omicron-S) variants. Similar to SARS-CoV-S, the binding of three nAbs on Delta-S induced partial dissociation of the trimeric S (*SI Appendix*, Fig. S6). We obtained a structure of the trimeric Delta-S:X01:X10:X17 at a resolution of 3.54 Å (global refinement) and performed localized refinement focusing on the interface and achieved a structure at 3.77 Å resolution (Fig. 2 *I*–*K* and *SI Appendix*, Figs. S6 and S8 and Table S2). As for the Omicron variant, although X01 and X10 showed significantly decreased efficacies in both the binding and neutralizing assays (Fig. 1 *G* and *H*), we still obtained a medium-resolution (6.56 Å) structure of the Omicron-S:X01:X10:X17 immune complex (Fig. 2*L* and *SI Appendix*, Figs. S7 and S8 and Table S2).

The footprints of X01, X10, and X17 contained 18, 21, and 16 RBD residues respectively, with only the X10 footprint found to partially overlap with the ACE2-binding site (Y449, Q493) (Fig. 2 *M*–*O*). The footprints of three nAbs were nonoverlapping and therefore allowed for the simultaneous binding of all three nAbs to the RBD (Fig. 2 *M*–*P*). Furthermore, the footprint of X17 excluded all of the previous VOC and VOI mutations—those associated with the Alpha, Beta, Gamma, Delta, and Omicron variants (Fig. 2 *O*–*Q*). Similarly, the footprints of X10 and X01 contained only four (N440, E484, and Q493 corresponding to Omicron; L452 corresponding to Delta) and two (S371 and S375 associated with Omicron) VOC mutation residues, respectively (Fig. 2 *M* and *N*). These findings demonstrate the high conservation of epitopes among SARS-CoV-2 VOCs and thus the potential benefit of X17.

### Structural Basis for the Broad Neutralization of nAbs against VOCs.

**Fig. 3.**
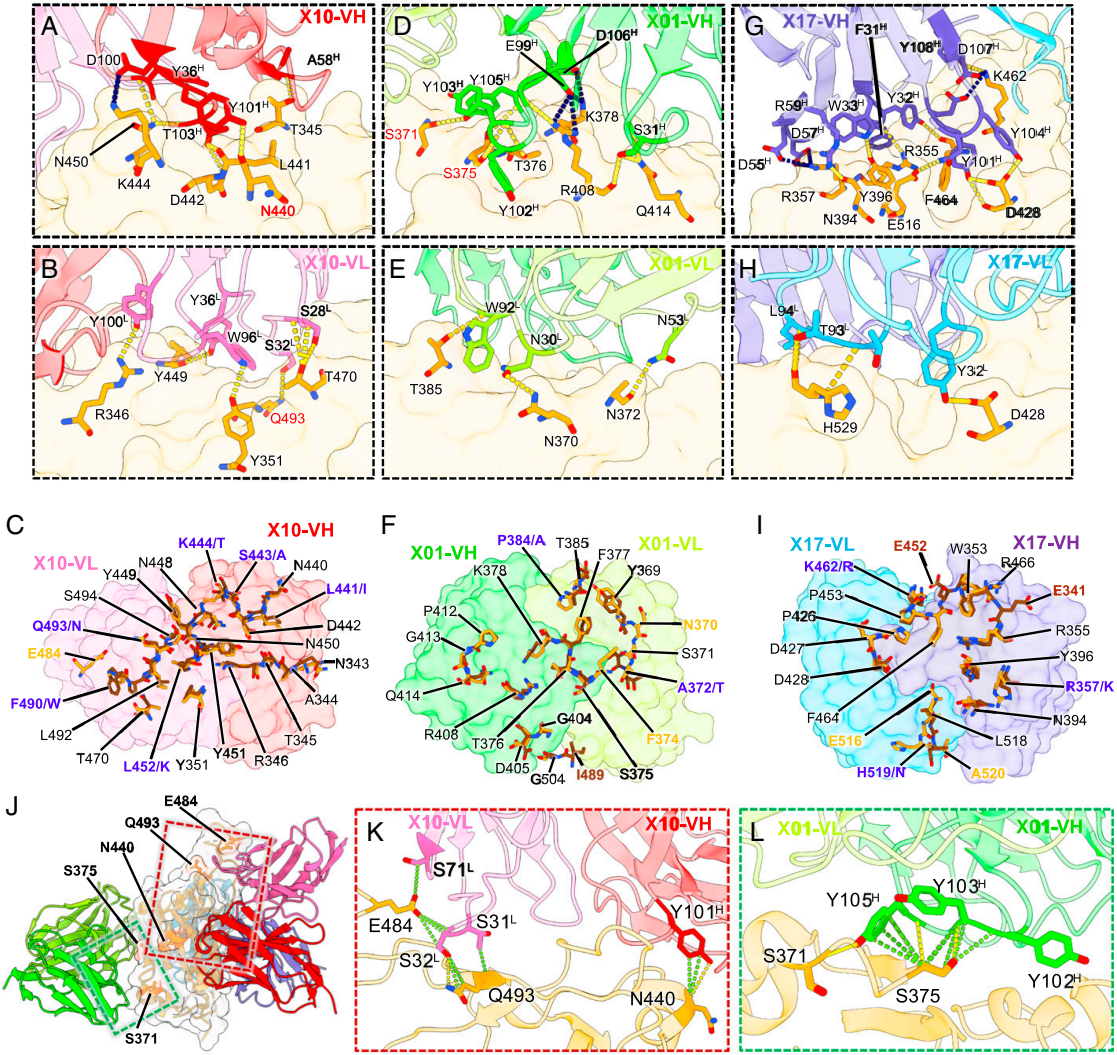
Interaction details of the three nAbs and structural basis for the decreased neutralization of X10 and X01 against the Omicron variant. (*A* and *B*) Interaction details of SARS-CoV-2 WT-RBD bound by X10. The X10 variable region of heavy chain (VH) (*A*) and variable region of light chain (VL) (*B*) mediate a network of hydrogen bonds (yellow dashed lines) and salt bridges (dark blue dashed lines). Models are shown as transparent cartoons. The RBD is also transparent. Residues that participate in hydrogen bonds or salt bridges are highlighted with the side chains shown. (*C*) Structural comparison of X10 epitopes on SARS-CoV-2 (orange stick) and SARS-CoV (brown stick). The shared residues of the binding sites on both RBDs are indicated in black (conserved residues between SARS-CoV-2 and SARS-CoV) and dark blue (non-conserved residues, residue abbreviations of SARS-CoV-2 and SARS-CoV are shown sequentially and separated by ‘/’), and the unique residues are shown in bold orange (SARS-CoV-2) and brown (SARS-CoV), respectively. (*D* and *E*) Interaction details of SARS-CoV-2 WT-RBD bound by X01 heavy (*D*) and light (*E*) chains. (*F*) Structural comparison of X01 epitopes on SARS-CoV-2 (orange stick) and SARS-CoV (brown stick). The VH (lime) and VL (yellow-green) of X01 are shown as transparent. (*G* and *H*) Interaction details of SARS-CoV-2 WT-RBD bound by the X17 heavy (*G*) and light (*H*) chains. (*I*) Structural comparison of the X17 epitopes on SARS-CoV-2 (orange stick) and SARS-CoV (brown stick). The VH (slate blue) and VL (sky blue) of X17 are shown as transparent surface representation. Residues on the RBDs involved in the interactions with three nAbs are labeled in black, and those diverse residues on epitopes of SARS-CoV and SARS-CoV-2 are highlighted. (*J*) Structure of SARS-CoV-2-S:X10:X01:X17 with highlighted residues (displayed in coral stick) on the WT-RBD with respect to the Omicron mutation (displayed in coral stick) that are involved in the nAb–RBD interaction. Three Fabs and RBD are shown in cartoon representation with a transparent surface. (*K* and *L*) Interaction details of X10 (*K*) and X01 (*L*) with residues involved in Omicron mutations. Contacts and hydrogen bonds are marked as green and yellow dashed lines, respectively.

We next analyzed the specific interaction details of three nAbs to SARS-CoV-2 WT, Delta, and SARS-CoV RBDs, respectively. The X10 epitope in its interaction with the WT RBD comprises 11 residues (R346, Y351, T345, N440, L441, D442, K444, Y449, N450, T470, and Q493) that form an extensive interaction network of 15 hydrogen bonds and one salt bridge (Figs. 2*M* and 3 *A* and *B*). Although the X10 epitope contains the VOC mutation site L452 (Fig. 2*M*), this residue does not participate in any hydrogen bonding or salt bridge interactions with X10 (Fig. 3*B*); however, the longer side chain of Arg formed by the L452R substitution in the Delta RBD instead leads to the formation of additional hydrogen bonds with X10 (*SI Appendix*, Fig. S10*B*), and this may explain why X10 confers a more than fivefold higher neutralization against the Delta variant (IC_50_: 33 ng/mL) than the D614G strain (IC_50_: 172 ng/mL) (Fig. 1*F* and *SI Appendix*, Table S1). In addition, 11 of the 20 residues of the X10 epitope in the SARS-CoV RBD are conserved among SARS-CoV-2 and SARS-CoV, with the remaining five residues harboring substitutions with the same class of hydrophilic amino acid (Figs. 2*Q* and 3*C*). Consequently, these substitutions lead to three hydrogen bonds in the interaction between the heavy chain of X10 and SARS-CoV RBD and seven hydrogen bonds and one salt bridge for X10 with the SARS-CoV-2 RBD (Fig. 3*A* and *SI Appendix*, Fig. S10*C*). Notably, compared with X10 and SARS-CoV-2 RBD binding, the interactions between the light chain of X10 and SARS-CoV RBD are enhanced by the presence of an additional hydrogen bond (eight vs. nine, respectively) and a salt bridge (zero vs. one, respectively) (Fig. 3*B* and *SI Appendix*, Fig. S10*D*). Therefore, although X10 recognizes diverse epitopes between SARS-CoV-2 and SARS-CoV, the mAb can still effectively bind to and neutralize SARS-CoV (Fig. 1*A* and *SI Appendix*, Table S1).

X01 recognizes a conserved epitope near the well-known CR3022 binding site (35) but closer to the ACE2 binding site (36) (Fig. 2*N* and *SI Appendix*, Fig. S11). Similar to X10, there is an elaborate interaction network of 15 hydrogen bonds and four salt bridges between X01 and SARS-CoV-2 RBD at the interface (Fig. 3 *D* and *E*). Similarly, X01 also strongly interacts with the Delta RBD and SARS-CoV RBD (*SI Appendix*, Fig. S10 *E–H*). The epitope of X01 on the SARS-CoV RBD comprises 17 residues, as compared with 18 residues in SARS-CoV-2 RBD, of which 14 residues are conserved (Figs. 2*Q* and 3*F*); this conservation accounts for the comparable neutralizing potencies of X01 against SARS-CoV and SARS-CoV-2 (Fig. 1*A* and *SI Appendix*, Table S1).

The X17 epitope locates to a cryptic site away from the ACE2-binding site, reminiscent of the reported sites for S2H97 (26, 37) and 6D6/7D6 (38) (Fig. 2*O* and *SI Appendix*, Fig. S11*A*). We found that X17 makes contact with the WT RBD primarily through nine residues (R355, R357, N394, Y396, D428, K462, F464, E516, H519) via 15 hydrogen bonds and five salt bridges (Fig. 3 *G* and *H*); this binding pattern is highly similar in X17 binding to Delta RBD and SARS-CoV RBD (*SI Appendix*, Fig. S10 *I–L*). In particular, 14 of the 16 residues of the epitope on SARS-CoV RBD are consistent with those on SARS-CoV-2 RBD, with the remaining two residues substituted by similar amino acids (R357K and K462R) (Figs. 2*Q* and 3*I*).

In general, class 5 nAb epitopes tend to be highly conserved among *Sarbecoviruses*, with the interaction site distant from most if not all of the mutation sites in SARS-CoV-2 VOCs, including the Omicron variant (26, 37) (Fig. 2 *O*–*Q* and *SI Appendix*, Fig. S11*B*). Thus, compared with X10 and X01, X17 is an optimal nAb, showing excellent binding activity to Omicron (EC_50_: 0.005 μg/mL), albeit with unsatisfactory neutralization levels (IC_50_: 1.67 μg/mL) (Fig. 1 *G* and *H*).

As for the Omicron variant, the E484, Q493, and N440 mutation sites are involved in its interaction with X10, whereas the S371 and S375 sites are involved in its interaction with X01 (Figs. 2*P* and 3*J*). We found that E484, Q493, and N440 on the WT RBD provide appreciable contact as well as two hydrogen bonding interactions with X10 (Fig. 3*K*). Although single mutations to any of these three residues has no effect or only slightly alters the binding of X10 (Fig. 1*D*), their synchronous mutation likely decreases the neutralization efficacy of X10; e.g., against the Omicron variant. Likewise, S371 and S375 in the WT RBD—residues that are also included in the X01 epitope—make appreciable interactions with X01 (Fig. 3 *J* and *L*). S371 and especially S375 contribute multiple contacts as well as hydrogen bonding interactions: S371 and Y105^H^, and S375 and both Y103^H^ and Y105^H^ (Fig. 3*L*). Therefore, we infer that mutations to these residues could dramatically weaken the interaction between X01 and RBD and, in turn, considerably decrease the neutralizing activity of X01 against Omicron (Fig. 1*H*). Therefore, the combination of multiple mutations in the Omicron RBD makes it easier to escape the cross-neutralizing antibodies X10 and X01, like most other existing cross-neutralizing antibodies (11). Considering the highly conserved epitope and broadly neutralizing breadth of the class 5 nAb X17, this antibody may serve as an essential component for the production of next-generation antibody mixture therapeutics against various SARS-CoV-2 variants in the future.

### Triple-Antibody mixture Resists Viral Escape In Vitro.

**Fig. 4.**
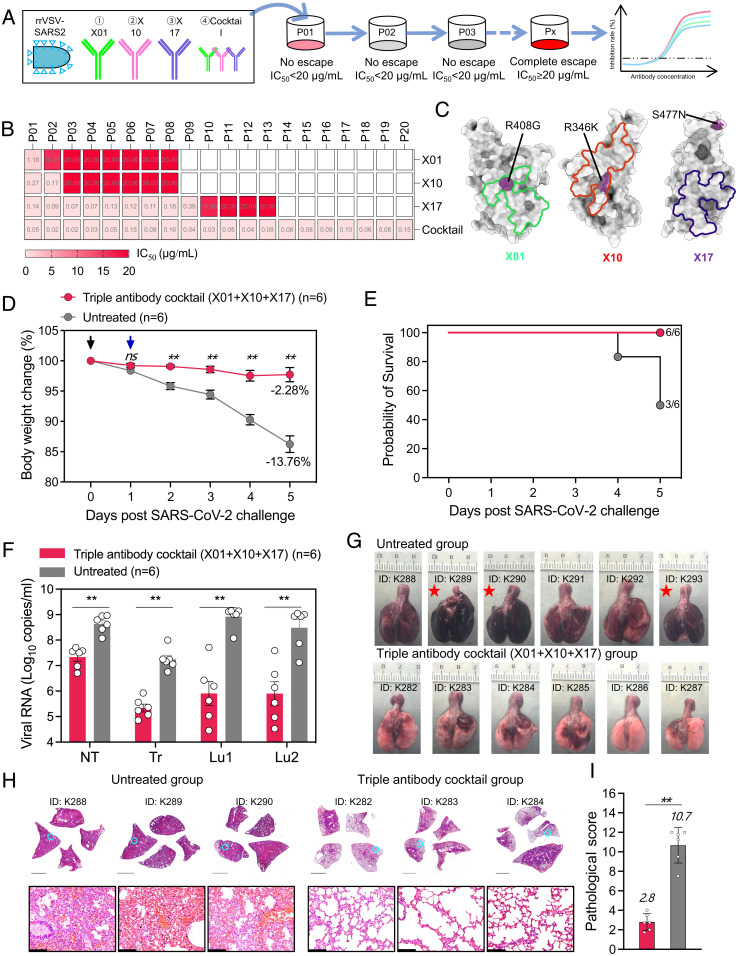
Efficacy of the triple-antibody mixture in resistance to escape mutation and protecting against SARS-CoV-2 B.1.351 infection in hamsters. (*A*) Viral escape evaluation scheme. (*B*) IC_50_ values of single antibodies (X01, X10, and X17) and the triple-antibody mixture against replicative rVSV-SARS2 prototype strain in different passages. Replicative rVSV-SARS2 variants in some passages that cause IC_50_ values >20 μg/mL are identified as ‘‘complete escape.’’ The IC_50_ values >20 μg/mL are uniformly calibrated as 20 μg/mL. (*C*) Representation of escape amino acid mutations on SARS-CoV-2 RBD. For X01, X10 and X17, the escape amino acid mutations are marked in green, red and purple, respectively. (*D*) Groups of six hamsters were intravenously administered (blue arrow) with a triple-antibody mixture (1:1:1 mixture of X01, X10, and X17 nAbs) at a total dosage of 35 mg/kg (red) or PBS (gray) as control at 1 d post B.1.351 intranasal infection (black arrow). Changes in body weight after infection are plotted. Mean weight loss for each group at 5 d post B.1.351 challenge is indicated. Data are mean ± SEM. (*E*) Kaplan–Meier survival plot. (*F*) Concentrations of viral RNA in the lysates of the nasal turbinate (NT), trachea (Tr), and lung regions proximal (Lu1) and distal (Lu2) to the hilum from hamsters were quantified. Data are shown as mean ± SEM. The difference between the groups was analyzed by Mann–Whitney *U* test. (*G*) Gross observations of lung tissues from untreated and triple-antibody mixture treated hamsters at 5 d post B.1.351 challenge. Red stars indicate hamsters that had died due to SARS-CoV-2 B.1.351. (*H*) H&E staining of four whole lung lobes collected from the PBS (untreated) group and the triple-antibody mixture group at 5 d post infection. *Top*, views of the four lobes (Scale bars, 4 mm). *Bottom*, circled (cyan) regions are enlarged (Scale bars, 124 μm). (*I*) Pathological severity scores for hamster lungs at 5 d post challenge. The average score for four independent lobes is calculated as the pathological severity score for each hamster. Data are shown as mean ± SEM. Differences between groups were determined by Mann–Whitney *U* test. Asterisks indicate statistical significance (**P < 0.01).

Previous studies have reported that combination therapy using dual nAbs that target noncompeting RBD epitopes decreases the propensity for rapid viral escape caused by monotherapy (13, 15, 29). To understand the escape characteristics of the SARS-CoV-2 under antibody selection pressure, we performed in vitro escape selection experiments using a previously reported replicative recombinant VSV-expressing S protein of the SARS-CoV-2 prototype strain (rVSV-SARS2) (29) (Fig. 4*A*). rVSV-SARS2 became resistant to X01 or X10 over three passages (Fig. 4*B* and *SI Appendix*, Fig. S12*A*), whereas X17 could maintain its neutralizing activity over 11 consecutive passages, with complete viral escape raised at passage 13 (P13). These findings suggest that the class 5 epitope is relatively more tolerant to immunologic pressure (Fig. 4*B* and *SI Appendix*, Fig. S12*A*). Notably, we found that the triple-antibody mixture showed no evidence of viral escape, even after 20 passages (Fig. 4*B* and *SI Appendix*, Fig. S12*A*), and the triple-antibody mixture also effectively inhibited the escape of the Omicron variant (*SI Appendix*, Fig. S12 *B* and *C*), indicating that this combination of three cross-neutralizing antibodies may benefit in preventing the rapid emergence of SARS-CoV-2 escape mutation.

Both the X01 and X10 induced the rapid escape driven by a single amino acid substitution of R408G and R346K, respectively, both of which are located in the footprints of two nAbs (Fig. 4*C* and *SI Appendix*, Fig. S13). In contrast, the X17 was resistant to rapid viral escape. The partial viral escape was detected at P10 when the mutation of S477N was present, and the complete viral escape was achieved at P11, resulting from the additional D614G substitution (Fig. 4*C* and *SI Appendix*, Fig. S13). This result highlights the inherent advantage of combination therapy of antibodies targeting different epitopes to avoid SARS-CoV-2 escape mutation.

### X10, X01, and ×17 Mixture Efficiently Protects Hamsters from Beta Variant Infection.

Considering the diverse epitopes, complementary neutralizing breadth, and resistance to the viral escape of X10, X01 and X17, we subsequently evaluated the therapeutic activity of these three antibodies as a triple-antibody mixture against infection with the Beta variant (B.1.351) in a Syrian hamster model. Following intranasal challenge with 1 × 10^4^ plaque-forming units (PFUs) of B.1.351, the antibody mixture was intravenously administered at a single dose of 35 mg/kg (each antibody at 11.7 mg/kg) at 1 d postinfection (dpi). Quantification of viral load and pathological analysis were carried out using respiratory tract samples at 5 dpi. Hamsters in the untreated group showed a significant loss in body weight (∼13.8%), with 50% of animals dead at 5 dpi (Fig. 4 *D* and *E*). In contrast, hamsters in the treated group maintained their weight levels (2.3% loss) and all survived to 5 dpi (Fig. 4 *D* and *E*). These findings suggest the therapeutic efficacy of the triple-antibody mixture.

Next, we measured the viral load in lung tissue samples to further evaluate the efficacy of antibody mixtures in the inhibition of viral replication at 5 dpi. Whereas the viral RNA load in the untreated group surged to approximately 1 × 10^9^ copies/mL in lung tissues, including lung regions proximal (Lu1) and distal (Lu2) to the hilum, the load in the antibody-treated group was reduced two to three orders of magnitude (Fig. 4*F*). Moreover, there was a significantly reduced viral load in nonlung respiratory tracts, such as the nasal turbinates and trachea among hamsters treated with the antibody mixture, as compared with untreated hamsters (Fig. 4*F*).

Viral infection–related lung damage was further evaluated. In gross observations of lung tissues, we found that the antibody mixture effectively inhibited the formation of multifocal diffuse hyperemia and consolidation as compared with the untreated group (Fig. 4*G*). In addition, through histopathological examination, we observed no significant lesions to alveolar epithelial cells or focal hemorrhage in the lung tissues of hamsters in the antibody-treated group as compared with the untreated group (Fig. 4*H* and *SI Appendix*, Fig. S14). Treatment with the antibody mixture thus profoundly decreased the mean pathological severity score to 2.8 as compared with 10.7 for the untreated group (Fig. 4*I*). Collectively, these results suggest that the triple-antibody mixture can effectively protect hamsters against infection with the Beta variant as well as any subsequent infection-related lung damage.

## Discussion

The emerging SARS-CoV-2 VOCs, particularly the Omicron variant, have shown increased transmissibility and resistance to antibody neutralization and thus increased the need for the development of broad-acting antibody therapeutics and vaccines (11, 12, 37). Various agents have been granted EUA by the U.S. Food & Drug Administration for treatment of COVID-19. However, this authorization was recently withdrawn for the mAb bamlanivimab, developed by AbCellera Biologics and Eli Lilly, following evidence that the epitopes located on or adjacent to the RBM are highly subject to mutation under selection pressure. Indeed, even its use in combination with another noncompeting nAb (e.g., estevimab) was ineffective in recovering the neutralization potency of bamlanivimab against the SARS-CoV-2 P.1 variant, which emerged before the Omicron variant (39). RBD-specific antibodies can be categorized into at least five classes (classes 1–5) based on their binding modes and the competition with ACE2 (26). Class 3, class 4, and class 5 nAbs generally represented S309 (27), S2X259 (28), and S2H97 (26, 37), respectively (*SI Appendix*, Fig. S11*B*), showing broadly neutralizing breadths against many SARS-CoV-2 VOCs as well as SARS-CoV. However, most of the above nAbs, including those authorized under EUA, have quite a drastically reduced neutralizing activity against the Omicron variant (11, 12, 37, 40, 41). Consistently, the cross-neutralizing antibodies obtained in this study—X01, X10, and X17—also showed lowered or limited neutralizing potency against Omicron. Based on the previous categorization strategy, the nAbs X10, X01, and X17 are most likely classified as class 3, class 4, and class 5, respectively (*SI Appendix*, Fig. S11*A*). The binding sites of these rare class 5 nAbs are spatially cryptic and tend to be highly conserved among *Sarbecoviruses*; this conservation was confirmed for X17, with high conservation noted between SARS-CoV-2 and SARS-CoV and no mutations for any of the VOCs, including the Omicron variant. Unfortunately, class 5 nAbs, including X17, tend to have less-potent neutralizing efficacies (IC_50_: 1∼10 μg/mL), and this can limit their clinical applicability. To optimize the neutralization potency of X17, we could seek to modify the X17 volume to strongly block RBD attachment to ACE2 (42, 43). Overall, our results show that both conserved and cryptic epitopes may serve as ideal targets for the development of next-generation broad vaccines against SARS-CoV-2 and its VOCs.

Class C1 antibodies that potently neutralize SARS-CoV and SARS-CoV-2 as well as most VOCs have been generated in abundance by the sequential immunization with VSV-SARS and VSV-SARS2. Indeed, we have previously shown that the combined immunization of coronavirus S proteins can effectively elicit cross-neutralizing antibodies (38), with sequential immunization able to produce cross-neutralizing antibodies targeting the more-conserved antigenic sites. The emergence of the Omicron variant eliminated the broad neutralization potential of many of the previously reported nAbs elicited from infection or immunization of a single virus strain, e.g., the SARS-CoV or SARS-CoV-2 prototype strain. Although the Omicron variant induced a substantial mutational leap on the RBD, here we showed that we could achieve efficient immune focus on the most-conserved epitopes (i.e., epitopes targeted by class 5 antibodies) via sequential immunization with VSV-SARS and VSV-SARS2. Yet as anticipated, these mutations in the RBD harbored by the Omicron variant significantly prohibited the class 3 and class 4 nAbs we generated from showing any neutralizing potency. Specifically, mutations to RBD residues E484, Q493, and N440 in the Omicron variant are likely responsible for the diminished binding of our class 3 nAb, X10. Other research has similarly shown that a G446S mutation in this site diminished the activity of another class 3 nAb, REGN10987, against the Omicron variant (44). As for our class 4 nAb, X01, mutations at residues S371 and S375 were likely responsible for Omicron escape; a similar phenomenon was shown for Ab H014 (45). Considering its unprecedented evasion from most nAbs, the Omicron variant should become an important consideration for sequential immunization practices, in addition to the prototypical SARS-CoV and SARS-CoV-2, to achieve more accurate immune focus.

Over the past two years, several antibody mixtures have been tested against COVID-19, with varying results, although antibody mixtures to date have only considered two antibodies simultaneously, not three. The present work shows the combination of three cross-neutralizing nAbs against SARS-CoV-2 infection in animals. A three-mAb mixture (atoltivimab, maftivimab, and odesivimab) was previously tested against the Ebola virus and was approved in 2020 (46).

In this study, X01, X10, and X17 were identified to effectively protect against B.1.351 infection in vivo. These results provide important insights into the feasibility of developing a triple-antibody mixture against COVID-19. Structural analyses revealed the existence of at least three noncompeting cross-neutralizing epitopes around the RBD. By simultaneously occupying these epitopes, the triple-antibody mixture shielded the majority of the flank, performing excellent or even synergistic neutralization against SARS-CoV-2 and its variants. X01 and X10 moderately blocked the interaction of ACE2 with the RBD to confer potent neutralization (IC_50_: 0.05–0.16 μg/mL), and this virus–receptor interference would be efficacious against SARS-CoV-2 and most VOCs. The third nAb, X17, with excellent binding affinity, targeted a highly conserved and cryptic epitope, with a binding manner similar to that observed for CR3022-like antibodies (35). It is surmised that this interaction may further neutralize the virus by destabilization of the S protein. Furthermore, although less potent when compared to the use of single nAbs against other VOCs, the triple-antibody mixture showed a synergetic neutralizing efficacy (IC_50_: 2.84 μg/mL) against the Omicron variant. This dosage, while high, is not unprecedented, with the clinical administration of antibody therapeutics frequently employing extremely large dosages. (For example, former U.S. President Donald J. Trump received antibody treatment for COVID-19 with a total dosage of 8 g [6].) This 2.84 μg/mL dose may also provide effective protection from the Omicron variant, and this will be the focus of future animal studies.

Others have shown a higher frequency of mutations in the RBM over the rest of the RBD (47). Here, the footprints of three nAbs had zero to few places of overlap with the ACE2 binding site, which may be of benefit in avoiding antibody-induced immune escape; indeed, X17 was more resistant to escape compared with X01 and X10, and the triple-antibody mixture based on X17 thus provides a stronger prevention against a selection of rapid escape mutation. Therefore, the triple-antibody combination should be a promising candidate for immunotherapy against pandemic SARS-CoV-2 VOCs.

In summary, we show that sequential immunization can achieve immune focus on conserved epitopes of SARS-CoV and SARS-CoV-2 and induce efficacious concentrations of bnAbs against SARS-CoV, SARS-CoV-2, and certain VOCs. The combination of three bnAbs—X01, X10, and X17—exhibited synergistic neutralizing activities, resistance to viral escape, and protection against disease caused by the SARS-CoV-2 Beta variant in hamsters. The structural basis for the neutralization breadth and potency of this triple-antibody mixture stems from its nonoverlapping pattern of binding. Overall, this study suggests a pivotal strategy for the development of antibody vaccine therapeutics against SARS-CoV-2 with an immune focus.

## Materials and Methods

### Ethics Statement.

BALB/c mice were purchased from Shanghai SLAC Laboratory Animal Co., Ltd. Hamsters (LVG Golden Syrian hamsters) were purchased from Charles River Laboratories. All experiments with infectious SARS-CoV-2 were performed in biosafety level 3 and animal biosafety level 3 facilities. Golden Syrian hamsters were raised in specific pathogen-free animal feeding facilities. All animal studies were carried out in strict accordance with the recommendations of the Guide for the Care and Use of Laboratory Animals. The mouse and hamster studies were conducted under the approval of the Institutional Animal Care and Use Committee of Xiamen University. All animal experiments were approved by the Medical Ethics Committee (SUCM2021-112).

### Cell Lines.

Vero-E6 (American Type Culture Collection [ATCC], CRL-1586), BHK21 (ATCC, CCL-10), SP2/0 (ATCC), and 293T (kindly gifted by Dr. Jiahuai Han) cells were maintained in high-glucose DMEM (Sigma-Aldrich) supplemented with 10% FBS (Gibco), penicillin (100 IU/mL), and streptomycin (100 μg/mL) in a 5% CO_2_ environment at 37 °C and passaged every 2 d. BHK21-hACE2 cells were developed by stable transfection of an hACE2-expressing plasmid through puromycin resistance selection. All cell lines used in this study were routinely tested for mycoplasma and found to be mycoplasma-free.

### Production of Pseudoviruses.

rVSVs expressing the SARS-CoV S (GenBank: AY278554.2) (termed as rVSV-SARS), the SARS-CoV-2 prototype strain S (GenBank: MN908947) (termed as rVSV-SARS2), SARS-CoV-2 VOCs S, or 55 SARS-CoV-2 S with different single point mutations were generated as previously described (33). Briefly, SARS-CoV or SARS-CoV-2 genes encoding for the S protein were cloned separately into the pCAG eukaryotic expression plasmid (Addgene). These genes had an 18–amino acid C-terminal truncation. rSARS-CoV and rSARS-CoV2 were rescued by VSVdG-EGFP-G (Addgene, 31842) from the Vero E6 cells transfected with plasmids pCAG-SARS1-Sdel18 and pCAG-SARS2-Sdel18 (48), respectively. Supernatants were harvested and purified by Capto Core 700 (Cytiva) multimodal chromatography. The viral particles were collected in the column flowthrough.

### Neutralization Assay Based on VSV Pseudovirus.

Neutralizing activities of antibodies against WT SARS-CoV, WT SARS-CoV-2, WT SARS-CoV-2 VOCs, and SARS-CoV-2 bearing different single-point mutations were quantified based on rVSVs, as previously described (33). A series of diluted monoclonal antibodies was mixed with pseudoviruses carrying gene encoding for the S protein of SARS-CoV, SARS-CoV-2, or its variants, and incubated at 37 °C for 1 h, respectively. The mixture was then transferred to BHK21-hACE2 cells seeded in a 96-well microplate and incubated for 12 h. Fluorescence images were captured by Opera Phenix (PerkinElmer) and quantitatively analyzed by the Columbus system (PerkinElmer). The percentage reduction of EGFP in each well as compared with the control wells was calculated. IC_50_ values were determined by the four-parameter logistic regression using GraphPad Prism (version 8.0.1).

### Sequential Immunization and Cross-Neutralizing Antibody Screening.

BALB/c mice were immunized with purified rVSV-SARS and rVSV-SARS2 once a week, alternately. After six doses of immunization (three rounds of each), mice were killed and hybridomas were generated by fusing splenic cells with SP2/0 mouse myeloma cells. Hybridomas secreting cross-neutralizing antibodies against both SARS-CoV and SARS-CoV-2 were screened using a pseudovirus neutralization assay based on rVSV-SARS and rVSV-SARS2 as described previously (33); an inhibition rate of cell supernatant greater than 90% was the criterion for positive hybridoma clones. Positive hybridoma clones were expanded using a 3× limiting dilution and cultured in 75 cm^2^ flasks. mAbs were prepared by injecting hybridoma cells into the peritoneal cavities of pristine-primed BALB/c mice; ascitic fluid was collected after 9 to 12 d, and mAbs were purified from mouse ascites using protein A agarose columns (GE Healthcare).

### Amplification and Sequencing of Mouse Antibody Genes.

The amplification of mouse antibody genes was carried out as previously reported (49). Briefly, approximately 1 × 10^7^ hybridoma cells were obtained by centrifugation, with the pellet resuspended in 1 mL Trizol reagent (Invitrogen). Total RNA was extracted, dissolved in 20 μL of RNase-free water (Invitrogen), and stored at −80 °C for later use. RT-PCR was performed to amplify antibody heavy chain and light chain genes. The RT-PCR system consisted of 5 μL 10× buffer (Promega), 1 μL 2.5 mM dNTP Mix (Takara), 23 μL RNase-free water (Invitrogen), 0.2 μL AMV enzyme (Promega), 20 μL RNA product, and 1 μL each upstream and downstream primers. RT-PCR reactions were performed at 40 °C for 70 min. Finally, 50 μL cDNA was obtained. For PCR, 3 μL cDNA product was combined with 5 μL 10× buffer, 1 μL 2.5 mM dNTP Mix (Takara), 40 μL RNase-free water (Invitrogen), 0.5 μL rTaq enzyme (New England Biolabs), and 1 μL upstream and downstream primers. The PCR was carried out at 95 °C for 5 min, followed by 34 cycles of 95 °C for 40 s, 56 °C for 40 s, and 72 °C for 1 min, and finished with another 72 °C for 10 min. Finally, the amplification product of antibody heavy and light chain genes was recovered for sequencing. MEGA6 software (version 7.0) (https://www.megasoftware.net) was used for processing and analyzing gene sequences. The International Immunogenetics Information System database (https://www.imgt.org/) was used to compare and analyze the antibody heavy chain and light chain gene sequences and identify the functional regions of the antibody heavy and light chains.

### Blocking Capacity of nAbs against ACE2 Binding.

Microplates precoated with recombinant antigens of RBD were provided by Wantai BioPharm. Antibodies at 100 µg/mL were fivefold serially diluted, added to the wells (100 µL), and incubated at 37 °C for 0.5 h. ACE2-hFc (Wantai BioPharm) was diluted at 85 ng/mL in SD-1 (Wantai BioPharm), added to the wells (100 µL), and incubated at 37 °C for 30 min. The wells were then washed and incubated for 30 min with HRP-labeled goat anti-human antibody (Abcam) diluted at 1:5,000. Wells were washed again, and the reaction was catalyzed using *o*-phenylenediamine substrate at 37 °C for 10 min. The optical density at 450 nm wavelength (OD_450nm_) (reference, OD_630nm_) was measured on a Tecan microplate reader with a cutoff value of 0.1. The blocking capacity was measured quantitatively by comparing OD in the presence and absence of nAbs and transformed using the following equation: [1 - (OD_present_/OD_absent_)] × 100%. Blocking IC_50_ values were calculated by Prism software using nonlinear regression (four parameters).

### Competitive Binding Assay.

A competitive binding assay was used to assess for competition in binding between antibodies. Briefly, unlabeled nAbs (50 μg per well) or 20 mM PBS (Gibco) were added to SARS-CoV-2 RBD-coated 96-well microplates and incubated for 30 min at 37 °C. Next, HRP-conjugated nAbs were added at selected dilutions (OD readings ∼1.5). After incubation for 30 min at 37 °C, the microplates were rinsed and the color was developed. The blocking rate was measured quantitatively by comparing OD values in the presence and absence of competitor mAbs and transformed using the following equation: [1 - (OD_present_/OD_absent_)] × 100%.

### Cryo-EM Sample and Data Collection.

Aliquots (3 μL) of 3.5 mg/mL mixtures of purified SARS-CoV-2 WT-S, Delta-S, and Omicron-S proteins (Sino Biological Inc.) and SARS-CoV-S protein (Sino Biological Inc.) in complex with excess Fab fragments of the three nAbs were incubated in 0.01% (vol/vol) digitonin (Sigma) and then loaded onto glow-discharged (60 s at 20 mA) holey carbon Quantifoil grids (R1.2/1.3, 200 mesh, Quantifoil Micro Tools) using a Vitrobot Mark IV (ThermoFisher Scientific) at 100% humidity and 4 °C. Data were acquired using the SerialEM software on an FEI Tecnai F30 transmission electron microscope (ThermoFisher Scientific) operated at 300 kV and equipped with a Gatan K3 direct detector. Images were recorded in the 36-frame movie mode at a nominal 39,000× magnification at superresolution mode with a pixel size of 0.389 Å. The total electron dose was set to 60 e^−^ Å^−2^ and the exposure time was 4.5 s.

### Image Processing and 3-Dimensional Reconstruction.

Drift and beam-induced motion correction were performed with MotionCor2 (50) to produce a micrograph from each movie. Contrast transfer function fitting and phase-shift estimation were conducted with Gctf (51). Micrographs with astigmatism, obvious drift, or contamination were discarded before reconstruction. The following reconstruction procedures were performed using Cryosparc V3 (52). In brief, particles were automatically picked using “blob picker” or “Template picker.” Several rounds of reference-free 2-dimensional classifications were performed, and the selected good particles were then subjected to ab initio reconstruction, heterogeneous refinement, and final nonuniform refinement. The resolution of all density maps was determined by the gold-standard Fourier shell correlation curve, with a cutoff of 0.143 (53). Local map resolution was estimated with ResMap (54).

### Atomic Model Building, Refinement, and 3-Dimensional Visualization.

The initial models of the nAbs were generated from homology modeling by Accelrys Discovery Studio software (https://www.3dsbiovia.com). The structure of the SARS-CoV-2 RBD and SARS-CoV RBD from the structure of the WT trimeric S (PDB accession code 6VSB [55]) and SARS-CoV RBD in complex with antibody CR3022 (PDB accession code 7JN5 [56]), respectively, were used as the initial modes of our WT-RBD, Delta-RBD, and SARS-CoV RBD. We initially fitted the templates into the corresponding final cryo-EM maps using Chimera (57) and further corrected and adjusted them manually by real-space refinement in Coot (58). The resulting models were then refined with phenix.real_space_refine in PHENIX (59). These operations were executed iteratively until the problematic regions, Ramachandran outliers, and poor rotamers were either eliminated or moved to favored regions. The final atomic models were validated with Molprobity (60, 61). All figures were generated with Chimera or ChimeraX (62, 63).

The RBD from the structure of the WT trimeric S (PDB accession code 6VSB [55]) was used as the initial model in our WT-RBD and Omicron RBD. The initial models, template fitting, refinement, and validation were as described above.

### Generation of Replicative Recombinant VSV-SARS2 Virus.

Replicative recombinant VSV-SARS2 (rrVSV-SARS2) was generated by replacing the VSV glycoprotein with the native SARS-CoV-2 S protein from the Wuhan-Hu-1 strain (GenBank accession code MN908947) bearing a C-terminal 18–amino acid truncation and encoding for the GFP gene to be inserted at the 3′ end of the VSV genome. Next, 293T cells were plated on poly-L-lysine solution (Sigma-Aldrich)–treated plates and incubated overnight in DMEM (Sigma-Aldrich) containing 10% FBS (Gibco) and 1% penicillin/streptomycin/L-glutamine (Invitrogen). The following day, cells were infected by recombinant vaccinia virus producing the T7 RNA polymerase (rVV-T7) and transfected with the VSV genomic clone driven by a T7 promoter and helper plasmids expressing the VSV-N, VSV-P, VSV-G, and VSV-L with lipofectamine LTX reagent (Invitrogen). After 48 h, the supernatants of the transfected cells were cocultured with Vero E6 cells (ATCC) transfected with VSV-G. Cells were monitored for GFP expression or cytopathic effect (CPE) indicative of virus replication. Viruses were then expanded and titrated in BHK21-hACE2 cells. After collection, stocks of both viruses were clarified by centrifugation at 3,500 rpm for 5 min and then frozen at −80 °C.

### In Vitro Escape Studies.

Escape studies were performed with the rrVSV-SARS2 virus (29). Viral escape was selected by incubating rrVSV-SARS2 under antibody pressure ranging from 0.02 μg/mL to 20 μg/mL. After 60 min incubation, the mixture was used to infect 1 × 10^6^ Vero E6 cells at a multiplicity of infection of 1. Virus replication was monitored by screening for GFP expression or CPE over 96 h. When >90% cells were GFP-positive or exhibited 90 to 100% CPE, the supernatant was collected and clarified by centrifugation. For subsequent rounds of selection, 100 μL supernatant containing the virus was passaged under the same or greater antibody concentrations as in previous passages until complete CPE was observed after antibody treatment at a concentration of ≥20 μg/mL. Viruses from consecutive passages were then expanded and titrated in BHK21-hACE2 cells. Neutralization assays of antibodies against the consecutively passaged virus were made as previously described (33).

### Therapeutic Effects against the Beta Variant in Hamsters.

The therapeutic effects of the cross-neutralizing antibody mixture against the Beta variant (GISAID: EPI_ISL_2779639) were evaluated using a hamster model, as previously described (64). Briefly, hamsters were intranasally inoculated with 1 × 10^4^ PFU/100 µL SARS-CoV-2 Beta strain. The triple-antibody mixture composed of X01, X10, and X17 in a ratio of 1:1:1 was administrated intraperitoneally at a total dose of 35 mg/kg at 24 h postchallenge, with PBS used as a negative control. Health and body weight change were recorded daily for each rodent. Hamsters were killed at 6 d postchallenge for the detection of viral load in respiratory tract organs and for a pathogenic analysis of lung lobes. Indicators for therapeutic efficacy included body weight change, tissue viral RNA load, and histopathology examination score.

### Histopathological Studies.

For histopathological analyses, lung tissues were fixed in formalin for more than 48 h, dehydrated, and then embedded in paraffin wax. Sections (4 μm) were cut for histology and pathological staining according to routine histological practice. Hematoxylin and eosin (H&E) staining was employed to assess for the presence of lung pathogenic lesions, including pulmonary edema, consolidation, and inflammation. The standards for pathological scoring of lung tissues were derived from our previous study in a hamster model. Comprehensive pathological scores for each individual lung lobe were performed according to the lesion extent, e.g., alveolar septum hyperplasia, consolidation and impairment of alveolar structure, fluid exudation, mucus suppository, thrombus, inflammation recruitment, and infiltration of immune cells.

For each hamster, the pathological score was comprehensively evaluated using three or four lung lobes. In brief, H&E staining was used to analyze the severity of pathological change to each lung lobe. Pathological changes, as noted by 1) alveolar septal thickening and consolidation; 2) hemorrhage, exudation, pulmonary edema, and mucus; and/or 3) the recruitment and infiltration of inflammatory immune cells, were scored with respect to severity as follows: 0, no pathological change observed; 1, moderate pathological change; 2, mild pathological change; 3, severe pathological change; and 4, very severe pathological change. The average comprehensive pathological score for lung lobes was used to evaluate the severity of lung pathogenesis. The images of whole lung lobes were screened using EVOS M7000 high-throughput screening microscopy (Thermo Fisher Scientific).

### SARS-CoV-2 RNA Quantification.

Lung, tracheal, and nasal turbinate tissue samples were harvested from infected hamsters and homogenized with a TissueLyser II (Qiagen). SARS-CoV-2 RNA was extracted using the QIAamp Viral RNA Mini Kit ((52906, Qiagen). Viral RNA concentration was quantified using a SARS-CoV-2 RT-PCR kit (WS-1248, Wantai BioPharm) according to the manufacturer’s instructions.

### Quantification and Statistical Analysis.

GraphPad Prism (version 8.0.1) was used for all statistical calculations. The Mann–Whitney *U* test was used to compare continuous variables between groups. *P* values less than 0.05 were considered statistically significant; ns: not significant; *P < 0.05; **P < 0.01; ***P < 0.001, which are indicated in the legends of Fig. 1 and Fig. 4. IC_50_ values were calculated by nonlinear regression analysis (log[agonist] vs. response – variable slope [four parameters]).

## Supplementary Material

Supplementary File

## Data Availability

The cryo-EM density maps have been deposited in the Electron Microscopy Data Bank (EMDB) (https://www.ebi.ac.uk/emdb/) with the accession codes EMD-33047 (65) (SARS-CoV-2-S:X01:X10:X17), EMD-33050 (66) (Delta-S:X10:X01:X17), EMD-33048 (67) (Delta-S:X10:X01:X17 interface), EMD-33051 (68) (SARS-CoV-S:X10:X01:X17), EMD-33049 (69) (SARS-CoV-S:X10:X01:X17 interface), and EMD-33052 (70) (Omicron-S:X10:X01:X17), and the corresponding atomic coordinates have been deposited in the Protein Data Bank (https://www.rcsb.org) with the accession codes 7X7T (71) (SARS-CoV-2-S:X01:X10:X17), 7X7V (72) (Delta-S:X10:X01:X17), and 7X7U (73) (SARS-CoV-S:X10:X01:X17).

## Change History

October 6, 2022: Figure 2 has been updated; please see accompanying Correction for details.

## References

[1] P. Zhou , A pneumonia outbreak associated with a new coronavirus of probable bat origin. Nature 579, 270–273 (2020). 32015507 10.1038/s41586-020-2012-7 PMC7095418

[2] T. Burki, Understanding variants of SARS-CoV-2. Lancet 397, 462 (2021). 33549181 10.1016/S0140-6736(21)00298-1 PMC7906644

[3] C. Wang, P. W. Horby, F. G. Hayden, G. F. Gao, A novel coronavirus outbreak of global health concern. Lancet 395, 470–473 (2020). 31986257 10.1016/S0140-6736(20)30185-9 PMC7135038

[4] C. O. Barnes , SARS-CoV-2 neutralizing antibody structures inform therapeutic strategies. Nature 588, 682–687 (2020). 33045718 10.1038/s41586-020-2852-1 PMC8092461

[5] B. Ju , Human neutralizing antibodies elicited by SARS-CoV-2 infection. Nature 584, 115–119 (2020). 32454513 10.1038/s41586-020-2380-z

[6] K. Thomas, G. Kolata, President Trump received experimental antibody treatment. *NY Times*, 2 October 2020, Section A, p. 14.

[7] Anonymous, An EUA for sotrovimab for treatment of COVID-19. Med. Lett. Drugs Ther. 63, 97–98 (2021). 34181630

[8] Anonymous, An EUA for bamlanivimab—A monoclonal antibody for COVID-19. JAMA 325, 880–881 (2021). 33306087 10.1001/jama.2020.24415

[9] Anonymous, Casirivimab and imdevimab (REGEN-COV) for post-exposure prophylaxis of COVID-19. Med. Lett. Drugs Ther. 63, 130–131 (2021). 34544100

[10] M. Dougan ; BLAZE-1 Investigators, Bamlanivimab plus etesevimab in mild or moderate Covid-19. N. Engl. J. Med. 385, 1382–1392 (2021). 34260849 10.1056/NEJMoa2102685 PMC8314785

[11] Y. Cao , Omicron escapes the majority of existing SARS-CoV-2 neutralizing antibodies. Nature 602, 657–663 (2022). 35016194 10.1038/s41586-021-04385-3 PMC8866119

[12] S. Cele , Omicron extensively but incompletely escapes Pfizer BNT162b2 neutralization. Nature 602, 654–656 (2022). 35016196 10.1038/s41586-021-04387-1 PMC8866126

[13] A. Baum , Antibody cocktail to SARS-CoV-2 spike protein prevents rapid mutational escape seen with individual antibodies. Science 369, 1014 (2020). 32540904 10.1126/science.abd0831 PMC7299283

[14] Z. M. Liu , Identification of SARS-CoV-2 spike mutations that attenuate monoclonal and serum antibody neutralization. Cell Host Microbe 29, 477 (2021). 33535027 10.1016/j.chom.2021.01.014 PMC7839837

[15] Y. Weisblum , Escape from neutralizing antibodies by SARS-CoV-2 spike protein variants. eLife 9, e61312 (2020). 33112236 10.7554/eLife.61312 PMC7723407

[16] P. F. Wang , Antibody resistance of SARS-CoV-2 variants B.1.351 and B.1.1.7. Nature 593, 130 (2021). 33684923 10.1038/s41586-021-03398-2

[17] M. Hoffmann , SARS-CoV-2 variants B.1.351 and P.1 escape from neutralizing antibodies. Cell 184, 2384 (2021). 33794143 10.1016/j.cell.2021.03.036 PMC7980144

[18] P. F. Wang , Increased resistance of SARS-CoV-2 variant P.1 to antibody neutralization. Cell Host Microbe 29, 747 (2021). 33887205 10.1016/j.chom.2021.04.007 PMC8053237

[19] Q. Q. Li , The impact of mutations in SARS-CoV-2 spike on viral infectivity and antigenicity. Cell 182, 1284 (2020). 32730807 10.1016/j.cell.2020.07.012 PMC7366990

[20] T. N. Starr, A. J. Greaney, A. S. Dingens, J. D. Bloom, Complete map of SARS-CoV-2 RBD mutations that escape the monoclonal antibody LY-CoV555 and its cocktail with LY-CoV016. Cell Rep Med 2, 100255 (2021). 33842902 10.1016/j.xcrm.2021.100255 PMC8020059

[21] M. Hoffmann , SARS-CoV-2 cell entry depends on ACE2 and TMPRSS2 and is blocked by a clinically proven protease inhibitor. Cell 181, 271 (2020). 32142651 10.1016/j.cell.2020.02.052 PMC7102627

[22] N. J. Matheson, P. J. Lehner, How does SARS-CoV-2 cause COVID-19? Science 369, 510–511 (2020). 32732413 10.1126/science.abc6156

[23] Y. Wu , A noncompeting pair of human neutralizing antibodies block COVID-19 virus binding to its receptor ACE2. Science 368, 1274 (2020). 32404477 10.1126/science.abc2241 PMC7223722

[24] L. Piccoli , Mapping neutralizing and immunodominant sites on the SARS-CoV-2 spike receptor-binding domain by structure-guided high-resolution serology. Cell 183, 1024 (2020). 32991844 10.1016/j.cell.2020.09.037 PMC7494283

[25] Y. Cao , Humoral immune response to circulating SARS-CoV-2 variants elicited by inactivated and RBD-subunit vaccines. Cell Res. 31, 732–741 (2021). 34021265 10.1038/s41422-021-00514-9 PMC8138844

[26] T. N. Starr , SARS-CoV-2 RBD antibodies that maximize breadth and resistance to escape. Nature 597, 97–102 (2021). 34261126 10.1038/s41586-021-03807-6 PMC9282883

[27] D. Pinto , Cross-neutralization of SARS-CoV-2 by a human monoclonal SARS-CoV antibody. Nature 583, 290–295 (2020). 32422645 10.1038/s41586-020-2349-y

[28] M. A. Tortorici , Broad sarbecovirus neutralization by a human monoclonal antibody. Nature 597, 103 (2021). 34280951 10.1038/s41586-021-03817-4 PMC9341430

[29] R. Copin , The monoclonal antibody combination REGEN-COV protects against SARS-CoV-2 mutational escape in preclinical and human studies. Cell 184, 3949–3961.e11 (2021). 34161776 10.1016/j.cell.2021.06.002 PMC8179113

[30] C. Shen , A multimechanistic antibody targeting the receptor binding site potently cross-protects against influenza B viruses. Sci. Transl. Med. 9, eaam5752 (2017). 29046433 10.1126/scitranslmed.aam5752

[31] D. M. Skowronski , Cross-lineage influenza B and heterologous influenza A antibody responses in vaccinated mice: Immunologic interactions and B/Yamagata dominance. PLoS One 7, e38929 (2012). 22745690 10.1371/journal.pone.0038929 PMC3382187

[32] A. Maroof, Y. M. Yorgensen, Y. Li, J. T. Evans, Intranasal vaccination promotes detrimental Th17-mediated immunity against influenza infection. PLoS Pathog. 10, e1003875 (2014). 24465206 10.1371/journal.ppat.1003875 PMC3900655

[33] H. L. Xiong , Robust neutralization assay based on SARS-CoV-2 S-protein-bearing vesicular stomatitis virus (VSV) pseudovirus and ACE2-overexpressing BHK21 cells. Emerg. Microbes Infect. 9, 2105–2113 (2020). 32893735 10.1080/22221751.2020.1815589 PMC7534347

[34] W. M. Zhao , The 2019 novel coronavirus resource. Yi Chuan 42, 212–221 (2020). 32102777 10.16288/j.yczz.20-030

[35] M. Yuan , Structural basis of a shared antibody response to SARS-CoV-2. Science 369, 1119–1123 (2020). 32661058 10.1126/science.abd2321 PMC7402627

[36] R. Yan , Structural basis for the recognition of SARS-CoV-2 by full-length human ACE2. Science 367, 1444–1448 (2020). 32132184 10.1126/science.abb2762 PMC7164635

[37] E. Cameroni , Broadly neutralizing antibodies overcome SARS-CoV-2 Omicron antigenic shift. Nature 602, 664–670 (2022). 35016195 10.1038/s41586-021-04386-2 PMC9531318

[38] T. Li , Cross-neutralizing antibodies bind a SARS-CoV-2 cryptic site and resist circulating variants. Nat. Commun. 12, 5652 (2021). 34580306 10.1038/s41467-021-25997-3 PMC8476643

[39] US Department of Health and Human Services. Update on COVID-19 variants and impact on bamlanivimab distribution. Available at: https://www.phe.gov/emergency/events/COVID19/investigation-MCM/Bamlanivimab/Pages/default.aspx. Accessed 22 April 2021.

[40] L. Liu , Striking antibody evasion manifested by the Omicron variant of SARS-CoV-2. Nature 602, 676–681 (2022). 35016198 10.1038/s41586-021-04388-0

[41] D. Planas , Considerable escape of SARS-CoV-2 Omicron to antibody neutralization. Nature 602, 671–675 (2022). 35016199 10.1038/s41586-021-04389-z

[42] Z. Ku , Nasal delivery of an IgM offers broad protection from SARS-CoV-2 variants. Nature 595, 718–723 (2021). 34082438 10.1038/s41586-021-03673-2 PMC8742224

[43] X. Ma , Nanoparticle vaccines based on the receptor binding domain (RBD) and heptad repeat (HR) of SARS-CoV-2 elicit robust protective immune responses. Immunity 53, 1315–1330.e9 (2020). 33275896 10.1016/j.immuni.2020.11.015 PMC7687490

[44] J. Hansen , Studies in humanized mice and convalescent humans yield a SARS-CoV-2 antibody cocktail. Science 369, 1010–1014 (2020). 32540901 10.1126/science.abd0827 PMC7299284

[45] Z. Lv , Structural basis for neutralization of SARS-CoV-2 and SARS-CoV by a potent therapeutic antibody. Science 369, 1505–1509 (2020). 32703908 10.1126/science.abc5881 PMC7402622

[46] U.S. Food & Drug Administration, FDA approves treatment for ebola virus. https://www.fda.gov/drugs/news-events-human-drugs/fda-approves-treatment-ebola-virus. Accessed 27 July 2022.

[47] D. Corti, L. A. Purcell, G. Snell, D. Veesler, Tackling COVID-19 with neutralizing monoclonal antibodies. Cell 184, 4593–4595 (2021). 34416148 10.1016/j.cell.2021.07.027 PMC8375150

[48] M. A. Whitt, Generation of VSV pseudotypes using recombinant ΔG-VSV for studies on virus entry, identification of entry inhibitors, and immune responses to vaccines. J. Virol. Methods 169, 365–374 (2010). 20709108 10.1016/j.jviromet.2010.08.006 PMC2956192

[49] S. Carbonetti , A method for the isolation and characterization of functional murine monoclonal antibodies by single B cell cloning. J. Immunol. Methods 448, 66–73 (2017). 28554543 10.1016/j.jim.2017.05.010 PMC5546949

[50] S. Q. Zheng , MotionCor2: Anisotropic correction of beam-induced motion for improved cryo-electron microscopy. Nat. Methods 14, 331–332 (2017). 28250466 10.1038/nmeth.4193 PMC5494038

[51] K. Zhang, Gctf: Real-time CTF determination and correction. J. Struct. Biol. 193, 1–12 (2016). 26592709 10.1016/j.jsb.2015.11.003 PMC4711343

[52] A. Punjani, J. L. Rubinstein, D. J. Fleet, M. A. Brubaker, cryoSPARC: Algorithms for rapid unsupervised cryo-EM structure determination. Nat. Methods 14, 290–296 (2017). 28165473 10.1038/nmeth.4169

[53] S. H. Scheres, S. Chen, Prevention of overfitting in cryo-EM structure determination. Nat. Methods 9, 853–854 (2012). 22842542 10.1038/nmeth.2115 PMC4912033

[54] A. Kucukelbir, F. J. Sigworth, H. D. Tagare, Quantifying the local resolution of cryo-EM density maps. Nat. Methods 11, 63–65 (2014). 24213166 10.1038/nmeth.2727 PMC3903095

[55] D. Wrapp , Cryo-EM structure of the 2019-nCoV spike in the prefusion conformation. Science 367, 1260–1263 (2020). 32075877 10.1126/science.abb2507 PMC7164637

[56] N. C. Wu , A natural mutation between SARS-CoV-2 and SARS-CoV determines neutralization by a cross-reactive antibody. PLoS Pathog. 16, e1009089 (2020). 33275640 10.1371/journal.ppat.1009089 PMC7744049

[57] E. F. Pettersen , UCSF Chimera—A visualization system for exploratory research and analysis. J. Comput. Chem. 25, 1605–1612 (2004). 15264254 10.1002/jcc.20084

[58] P. Emsley, K. Cowtan, Coot: Model-building tools for molecular graphics. Acta Crystallogr. D Biol. Crystallogr. 60, 2126–2132 (2004). 15572765 10.1107/S0907444904019158

[59] P. D. Adams , PHENIX: A comprehensive Python-based system for macromolecular structure solution. Acta Crystallogr. D Biol. Crystallogr. 66, 213–221 (2010). 20124702 10.1107/S0907444909052925 PMC2815670

[60] V. B. Chen , MolProbity: All-atom structure validation for macromolecular crystallography. Acta Crystallogr. D Biol. Crystallogr. 66, 12–21 (2010). 20057044 10.1107/S0907444909042073 PMC2803126

[61] X. Robert, P. Gouet, Deciphering key features in protein structures with the new ENDscript server. Nucleic Acids Res. 42, W320–W324 (2014). 24753421 10.1093/nar/gku316 PMC4086106

[62] T. D. Goddard , UCSF ChimeraX: Meeting modern challenges in visualization and analysis. Protein Sci. 27, 14–25 (2018). 28710774 10.1002/pro.3235 PMC5734306

[63] E. F. Pettersen , UCSF ChimeraX: Structure visualization for researchers, educators, and developers. Protein Sci. 30, 70–82 (2021). 32881101 10.1002/pro.3943 PMC7737788

[64] L. Yuan , Gender associates with both susceptibility to infection and pathogenesis of SARS-CoV-2 in Syrian hamster. Signal Transduct. Target. Ther. 6, 136 (2021). 33790236 10.1038/s41392-021-00552-0 PMC8009924

[65] H. Xiong , The neutralizing breadth of antibodies targeting diverse conserved epitopes between SARS-CoV and SARS-CoV-2. Electron Microscopy Data Bank https://www.ebi.ac.uk/emdb/EMD-33047. Deposited 17 August 2022. 10.1073/pnas.2204256119 PMC9407403 35972965

[66] H. Xiong , The neutralizing breadth of antibodies targeting diverse conserved epitopes between SARS-CoV and SARS-CoV-2. Electron Microscopy Data Bank https://www.ebi.ac.uk/emdb/EMD-33050. Deposited 17 August 2022. 10.1073/pnas.2204256119 PMC9407403 35972965

[67] H. Xiong , The neutralizing breadth of antibodies targeting diverse conserved epitopes between SARS-CoV and SARS-CoV-2. Electron Microscopy Data Bank https://www.ebi.ac.uk/emdb/EMD-33048. Deposited 17 August 2022. 10.1073/pnas.2204256119 PMC9407403 35972965

[68] H. Xiong , The neutralizing breadth of antibodies targeting diverse conserved epitopes between SARS-CoV and SARS-CoV-2. Electron Microscopy Data Bank https://www.ebi.ac.uk/emdb/EMD-33501. Deposited 17 August 2022. 10.1073/pnas.2204256119 PMC9407403 35972965

[69] H. Xiong , The neutralizing breadth of antibodies targeting diverse conserved epitopes between SARS-CoV and SARS-CoV-2. Electron Microscopy Data Bank https://www.ebi.ac.uk/emdb/EMD-33049. Deposited 17 August 2022. 10.1073/pnas.2204256119 PMC9407403 35972965

[70] H. Xiong , The neutralizing breadth of antibodies targeting diverse conserved epitopes between SARS-CoV and SARS-CoV-2. Electron Microscopy Data Bank https://www.ebi.ac.uk/emdb/EMD-33052. Deposited 17 August 2022. 10.1073/pnas.2204256119 PMC9407403 35972965

[71] H. Sun , Cryo-EM structure of SARS-CoV-2 spike protein in complex with three nAbs X01, X10 and X17. Protein data bank http://www.rcsb.org/pdb/explore/explore.do?structureId=7X7T. Deposited 10 March 2022.

[72] H. Sun , Cryo-EM structure of SARS-CoV spike protein in complex with three nAbs X01, X10 and X17. Protein data bank http://www.rcsb.org/pdb/explore/explore.do?structureId=7X7V. Deposited 10 March 2022.

[73] H. Sun , Cryo-EM structure of SARS-CoV-2 Delta variant spike protein in complex with three nAbs X01, X10 and X17. Protein data bank http://www.rcsb.org/pdb/explore/explore.do?structureId=7X7U. Deposited 10 March 2022.

