# Supplementary material for: The neutralizing breadth of antibodies targeting diverse conserved epitopes between SARS-CoV and SARS-CoV-2
Source: Proc Natl Acad Sci U S A. 2022 Aug 16;119(34):e2204256119. doi: 10.1073/pnas.2204256119 (PMC9407403; doi:10.1073/pnas.2204256119)
Supplement: Supplementary File [file pnas.2204256119.sapp.pdf]

Supplementary Information for

Neutralizing breadth of antibodies targeting diverse conserved epitopes between SARS-CoV and SARS-CoV-2

Hualong Xiong [1,#], Hui Sun [1,#], Siling Wang [1,#], Lunzhi Yuan [1,#], Liqin Liu [1,#], Yuhe Zhu [1,#], Jinlei Zhang [1,#], Yang Huang [1], Ruoyao Qi [1], Yao Jiang [1], Jian Ma [1], Ming Zhou [1], Le Ma [1], Rao Fu [1], Siping Yan [1], Yangtao Wu [1], Min Wei [1], Mingxi Yue [1], Yizhen Wang [1], Tingting Li [1], Zizheng Zheng [1], Hai Yu [1], Tong Cheng [1], Shaowei Li [1,*], Quan Yuan [1,*], Jun Zhang [1,*], Yi Guan [3,*], Qingbing Zheng [1,*], Tianying Zhang [1,*], Ningshao Xia [1,2,*]

[1] State Key Laboratory of Molecular Vaccinology and Molecular Diagnostics; National Institute of Diagnostics and Vaccine Development in Infectious Diseases, School of Public Health, School of Life Sciences, Xiamen University, Xiamen 361102, China

[2] Research Unit of Frontier Technology of Structural Vaccinology, Chinese Academy of Medical Sciences, Xiamen 361102, China

[3] State Key Laboratory of Emerging Infectious Diseases, University of Hong Kong, Hong Kong 999077, China.

* Corresponding authors: shaowei@xmu.edu.cn (S.L.), yuanquan@xmu.edu.cn (Q.Y.), zhangj@xmu.edu.cn (J.Z.), yguan@hku.hk (Y.G.), abing0811@xmu.edu.cn (Q.Z.), zhangtianying@xmu.edu.cn (T.Z.), nsxia@xmu.edu.cn (N.X.)

[#] These authors contributed equally.

This PDF file includes:

Supplementary Figures 1-14

Supplementary Tables 1-2

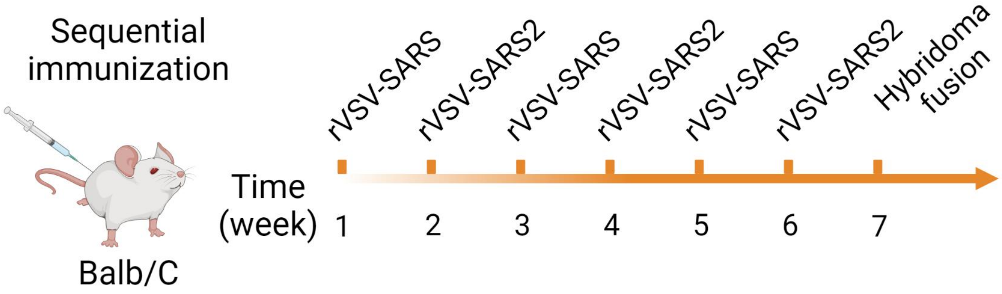

Fig. S1. Sequential immunization scheme. Mice were immunized six times with rVSV pseudoviruses carrying spike proteins of SARS-CoV and SARS-CoV-2, respectively, in order at interval of 1 week. After the immunization of three times of both rVSV-SARS and rVSV-SARS2, the hybridoma cell pools were screened for RBD-specific and cross-neutralizing mAbs at week 7. Figure was created by an online tool on *BioRender.com*.

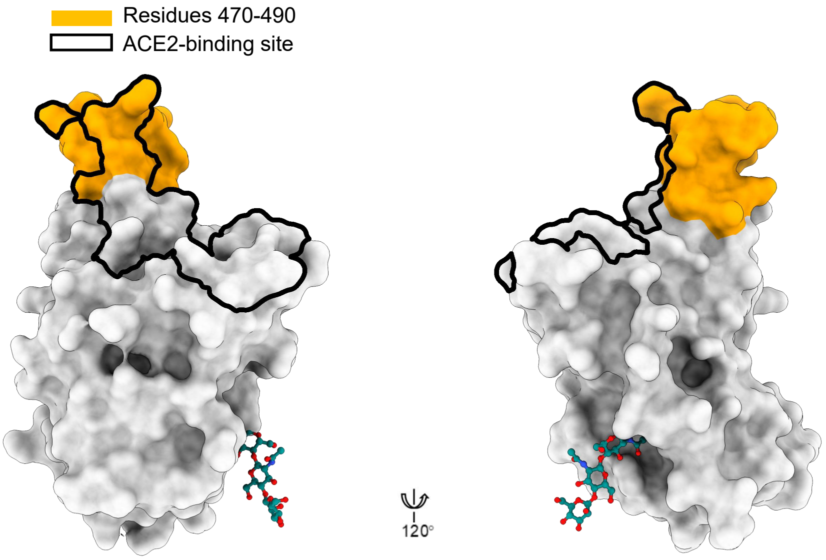

Fig. S2. Representation of residues 470-490 in SARS-CoV-2 RBD. RBD (PBD: 7R6W) is presented as surface (gray) representation with residues 470-490 colored in orange and ACE2-binding site highlighted by black line. The N343 glycan is shown as stick.

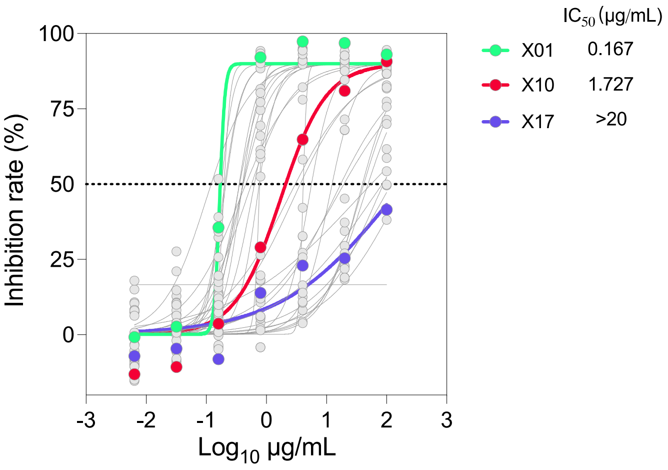

Fig. S3. Blocking activities of nAbs against the binding of ACE2 to spike protein. A total of 34 nAbs were tested and X01, X10 and X17 are indicated in magenta, cyan and green, respectively, and the blocking IC_{50} values were calculated by Prism software using non-linear regression (four parameters).

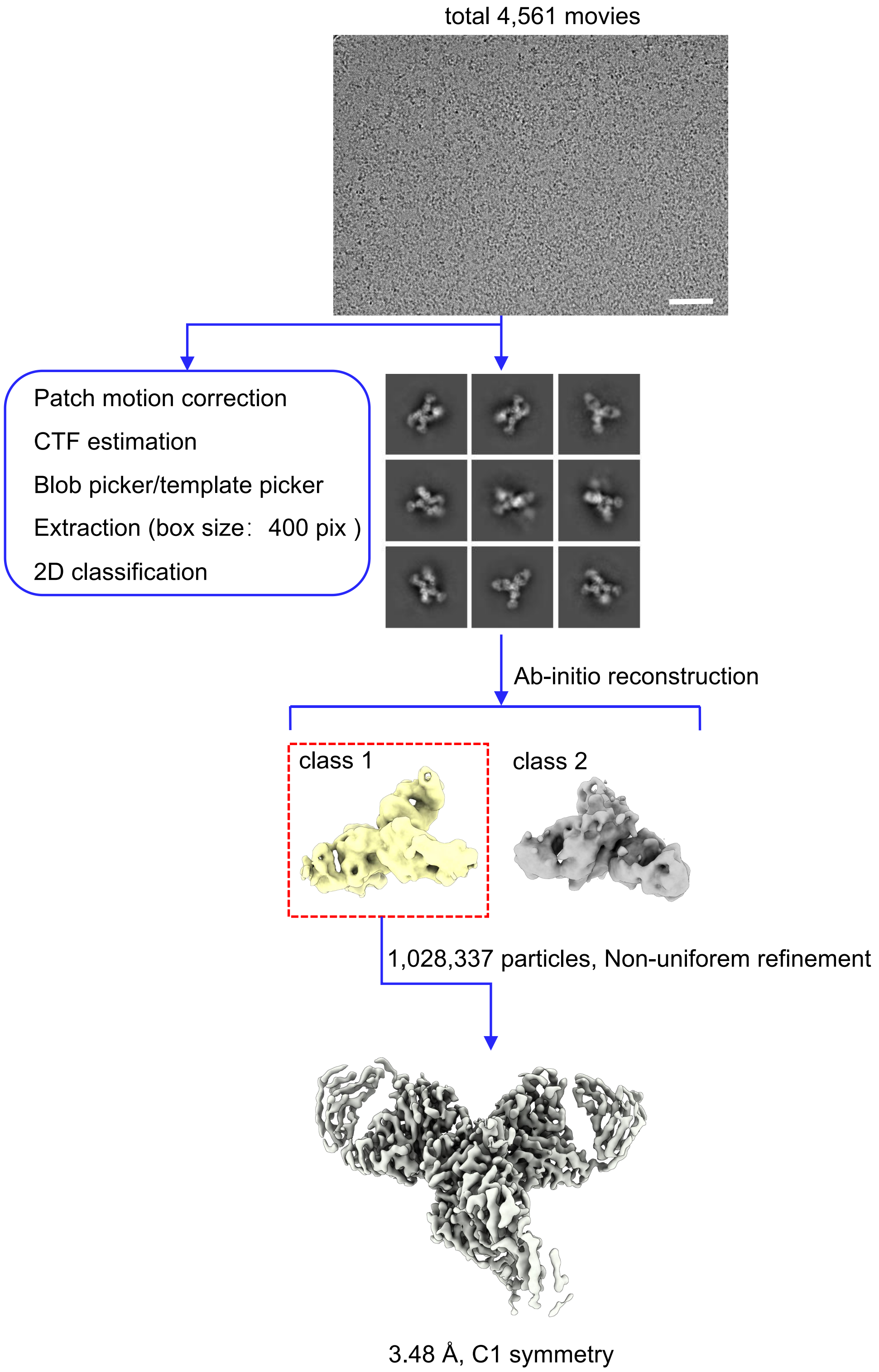

Fig. S4. Single-particle cryo-EM image processing workflow for the immune complex of SARS-CoV-2-S:X10:X01:X17. Representative electron micrograph, 2D classifications, initial models and final map are shown. Scale bar: 50nm.

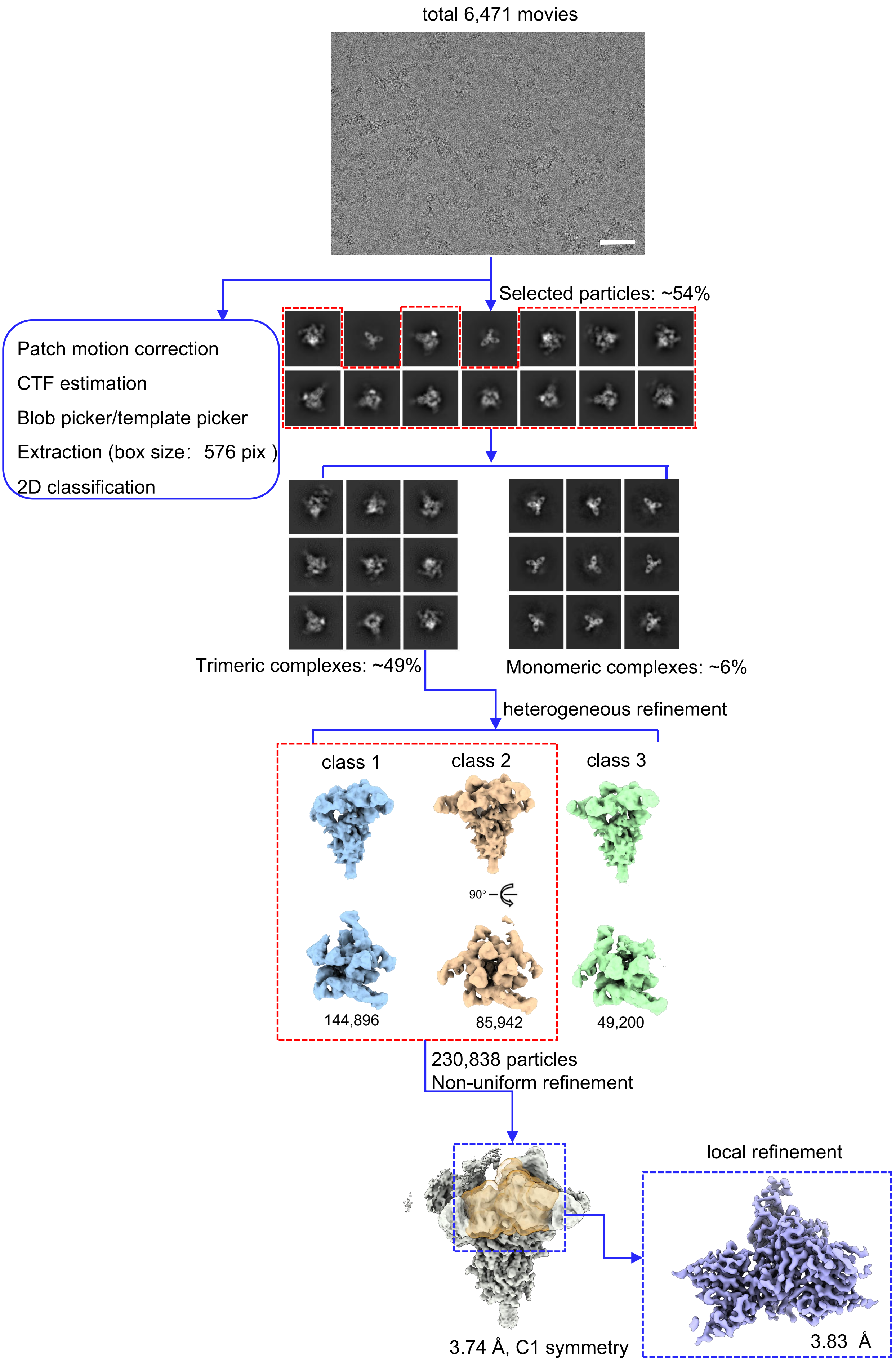

total 6,471 movies
Selected particles: ~54%
Patch motion correction
CTF estimation
Blob picker/template picker
Extraction (box size: 576 pix)
2D classification
Trimeric complexes: ~49%
Monomeric complexes: ~6%
heterogeneous refinement
class 1
class 2
class 3
90°
144,896
85,942
49,200
230,838 particles
Non-uniform refinement
local refinement
3.74 Å, C1 symmetry
3.83 Å

Fig. S5. Single-particle cryo-EM image processing workflow for the immune complex of SARS-CoV-S:X10:X01:X17. Representative electron micrograph, 2D classifications, heterogeneous refinement, non-uniform refinement and local refinement maps are shown. Scale bar: 50nm.

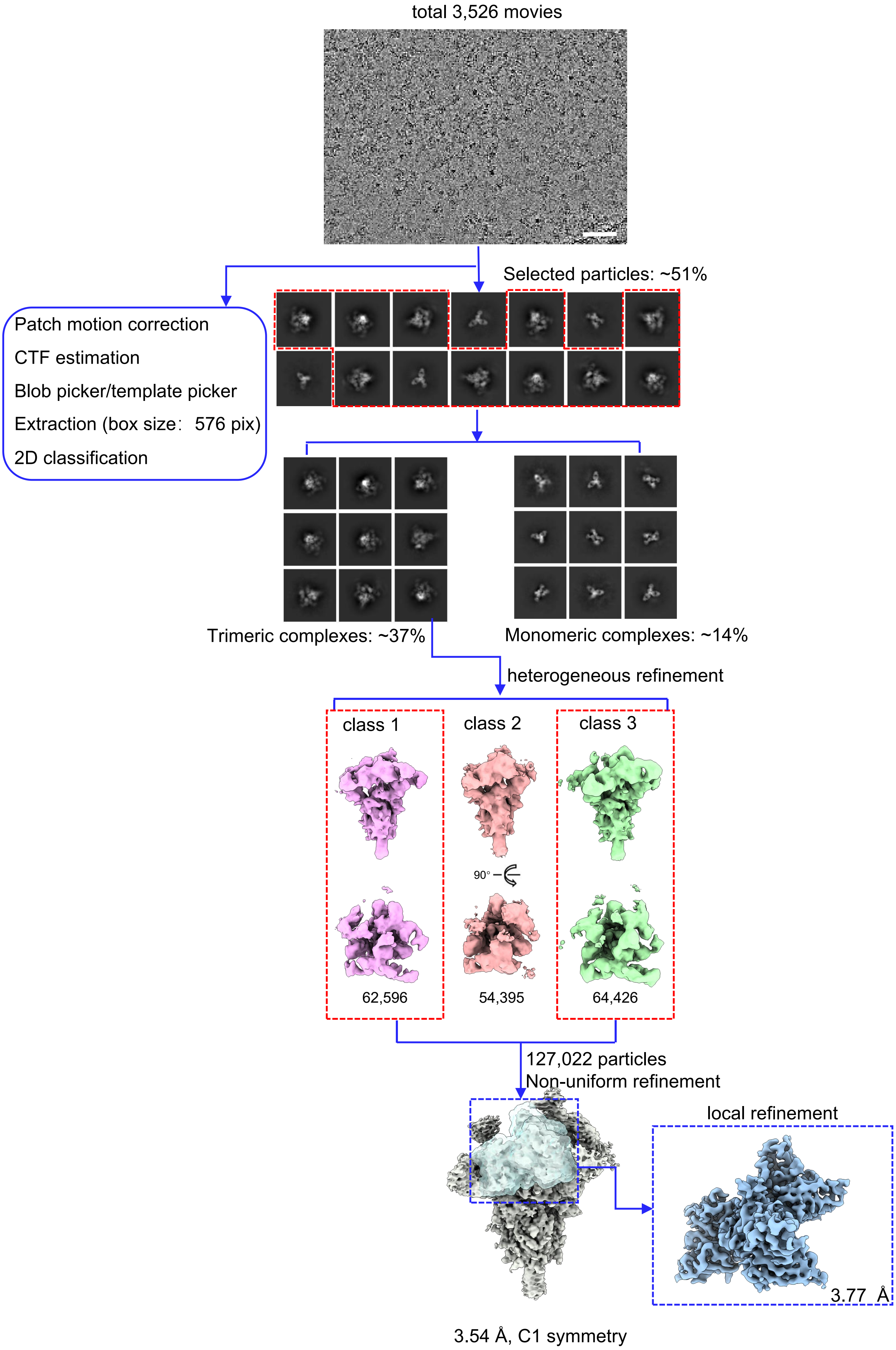
total 3,526 movies
Selected particles: ~51%
Patch motion correction
CTF estimation
Blob picker/template picker
Extraction (box size: 576 pix)
2D classification
Trimeric complexes: ~37%
Monomeric complexes: ~14%
heterogeneous refinement
class 1
class 2
class 3
90°
62,596
54,395
64,426
127,022 particles
Non-uniform refinement
local refinement
3.77 Å
3.54 Å, C1 symmetry

Fig. S6. Single-particle cryo-EM images processing workflow for the immune complex of Delta-S:X10:X01:X17. Representative electron micrograph, 2D classifications, heterogeneous refinement, non-uniform refinement and local refinement maps are shown. Scale bar: 50nm.

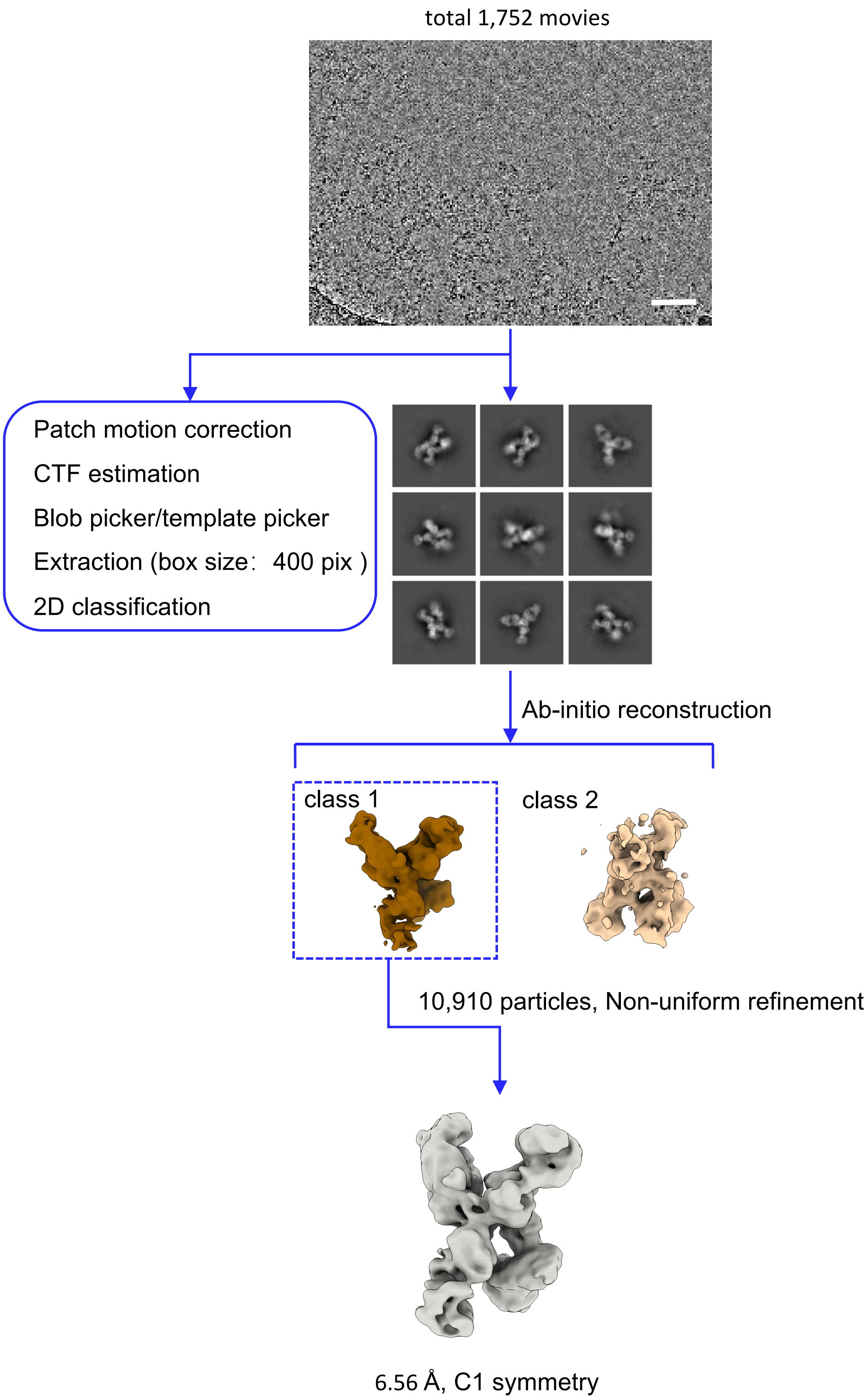

Fig. S7. Single-particle cryo-EM image processing workflow for the immune complex of Omicron-S:X10:X01:X17. Representative electron micrograph, 2D classifications, initial models and final map are shown. Scale bar: 50nm.

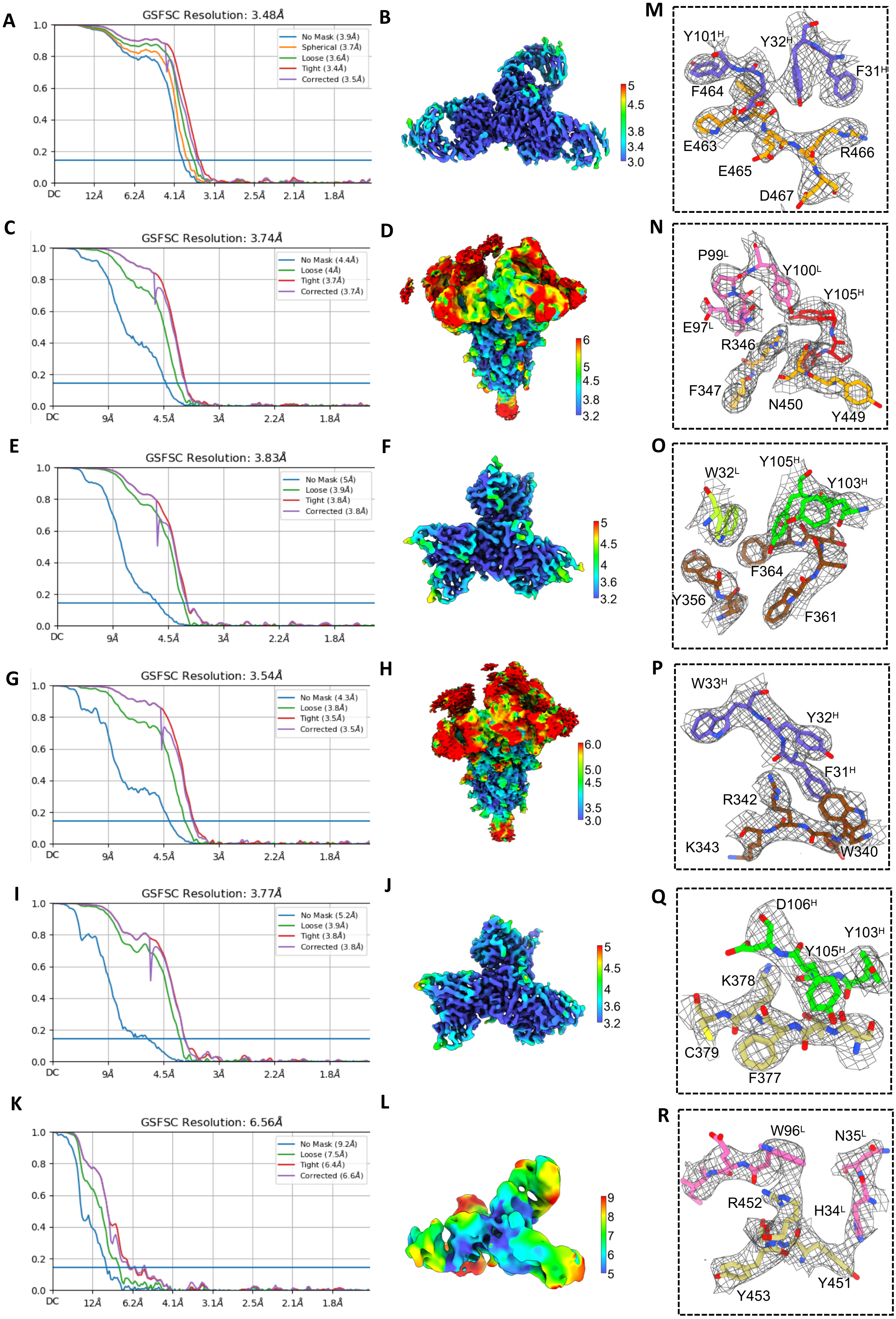
A
GSFSC Resolution: 3.48Å
No Mask (3.9Å)
Spherical (3.7Å)
Loose (3.6Å)
Tight (3.4Å)
Corrected (3.5Å)
DC 12Å 6.2Å 4.1Å 3.1Å 2.5Å 2.1Å 1.8Å
B
5 4.5 3.8 3.4 3.0
M
Y101H Y32H F31H F464 E463 R466 E465 D467
C
GSFSC Resolution: 3.74Å
No Mask (4.4Å)
Loose (4Å)
Tight (3.7Å)
Corrected (3.7Å)
DC 9Å 4.5Å 3Å 2.2Å 1.8Å
D
6 5 4.5 3.8 3.2
N
P99L Y100L Y105H E97L R346 F347 N450 Y449
E
GSFSC Resolution: 3.83Å
No Mask (5Å)
Loose (3.9Å)
Tight (3.8Å)
Corrected (3.8Å)
DC 9Å 4.5Å 3Å 2.2Å 1.8Å
F
5 4.5 4 3.6 3.2
O
W32L Y105H Y103H F364 Y356 F361
G
GSFSC Resolution: 3.54Å
No Mask (4.3Å)
Loose (3.8Å)
Tight (3.5Å)
Corrected (3.5Å)
DC 9Å 4.5Å 3Å 2.2Å 1.8Å
H
6.0 5.0 4.5 3.5 3.0
P
W33H Y32H F31H R342 K343 W340
I
GSFSC Resolution: 3.77Å
No Mask (5.2Å)
Loose (3.9Å)
Tight (3.8Å)
Corrected (3.8Å)
DC 9Å 4.5Å 3Å 2.2Å 1.8Å
J
5 4.5 4 3.6 3.2
Q
D106H Y103H Y105H K378 C379 F377
K
GSFSC Resolution: 6.56Å
No Mask (9.2Å)
Loose (7.5Å)
Tight (6.4Å)
Corrected (6.6Å)
DC 12Å 6.2Å 4.1Å 3.1Å 2.5Å 2.1Å 1.8Å
L
9 8 7 6 5
R
W96L N35L R452 H34L Y453 Y451

Fig. S8. Resolution estimation of the cryo-EM reconstructions and local density maps with atomics model. (A and B) Fourier shell correlation (FSC) curve (A) and local density map (B) of 3D reconstructions of SARS-CoV-2-S:X10:X01:X17 , (C and D) Fourier shell correlation (FSC) curve (C) and local density map (D) of global refinement of SARS-CoV:X10:X01:X17 (D), (E and F) Fourier shell correlation (FSC) curve (E) and local density map (F) of local refinement of SARS-CoV-RBD:X10:X01:X17, (G and H) Fourier shell correlation (FSC) curve (G) and local density map (H) of global refinement of Delta-S-:X10:X01:X17 (H), (I and J) Fourier shell correlation (FSC) curve (I) and local density map (J) of local refinement of Delta-RBD:X10:X01:X17, (K and L) Fourier shell correlation (FSC) curve (J) and local density map (L) of and Omicron-S:X10:X01:X17. (M-R) Representative density maps with corresponding models showing the interaction interfaces between antibodies and RBD of WT (M-N), SARS-CoV (O-P) and Delta variant(Q-R).

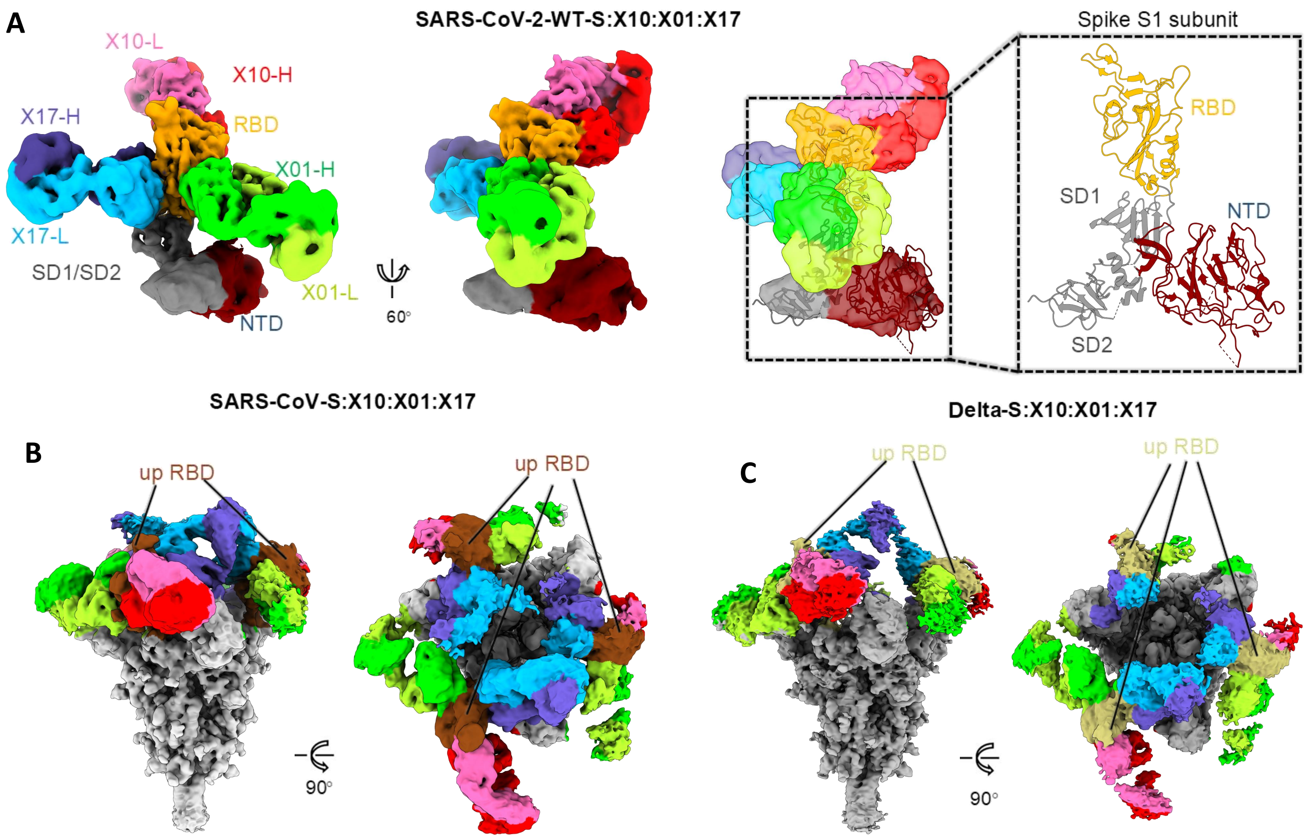

Fig. S9. Structural characterization of monomer (SARS-CoV-2) and trimericv (Delta and SARS-CoV) bound by three nAbs. (A) The domain colored cryo-EM map of SARS-CoV-2-S:X10:X01:X17 (left and middle). Transparency map (right) fitted with the model of S1-subunit of SARS-CoV-2 spike (PDB: 7S0E), the model is shown as cartoon. (B-C) The domain colored maps of Delta-S:X10:X01:X17 (B) and SARS-CoV-S:X10:X01:X17 (C). Each of up RBDs of Delta and SARS-CoV spike are bound by three Fabs but of three RBD in one trimer, only one RBD is saturated bound.

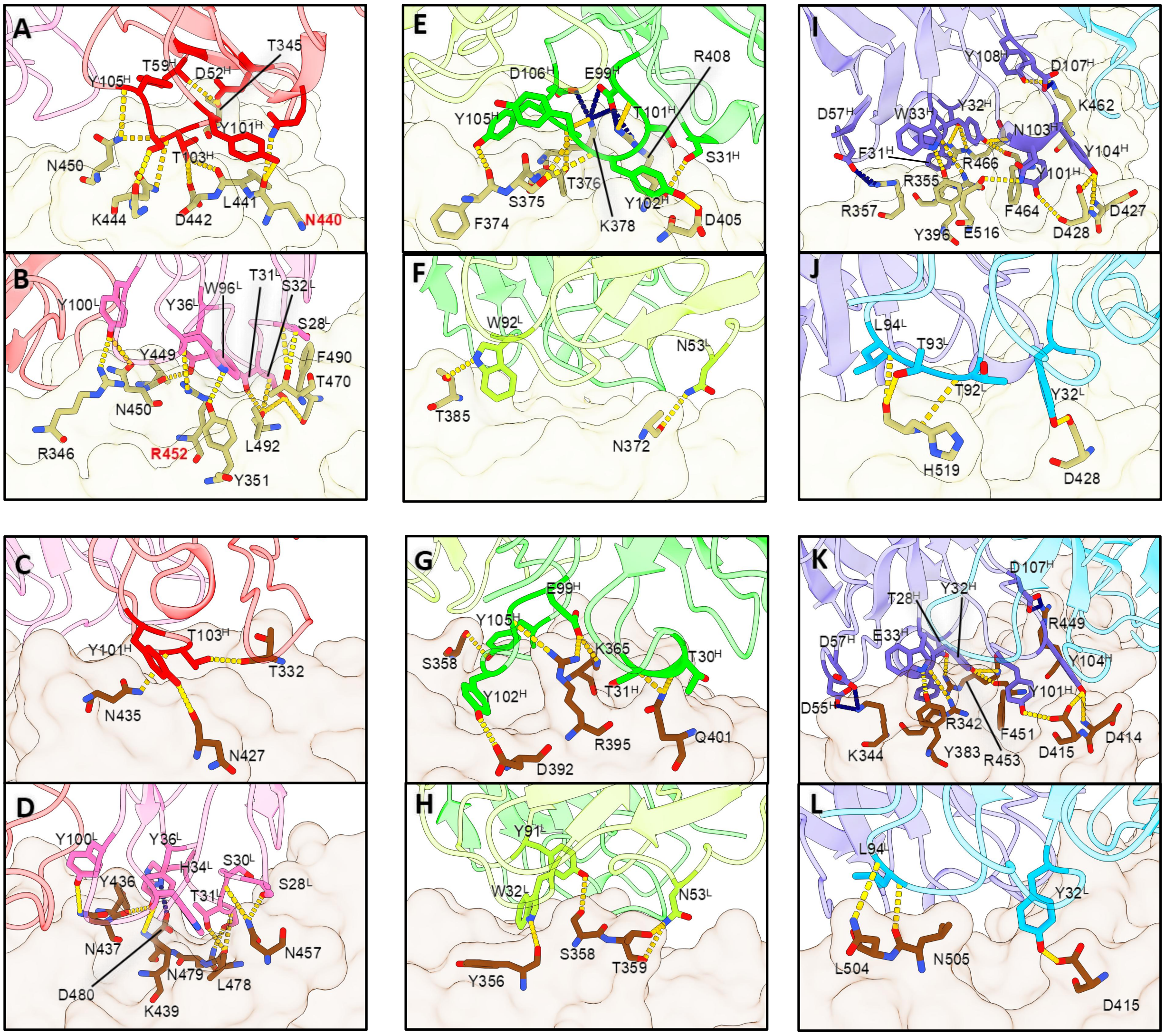

Fig. S10. Interactions details of Delta- and SARS-CoV-RBD bound by three Fabs. Close-up views of interactions details of Delta RBD bound by X10 (A, B), X01 (E, F) and X17 (I, J). Close-up views of interactions details of SARS-CoV RBD bound by X10 (C, D), X01 (G, H) and X17 (K, L). Yellow dash lines and dark blue dash lines indicates hydrogen bonds and salt-bridges between Fabs and RBD, respectively.

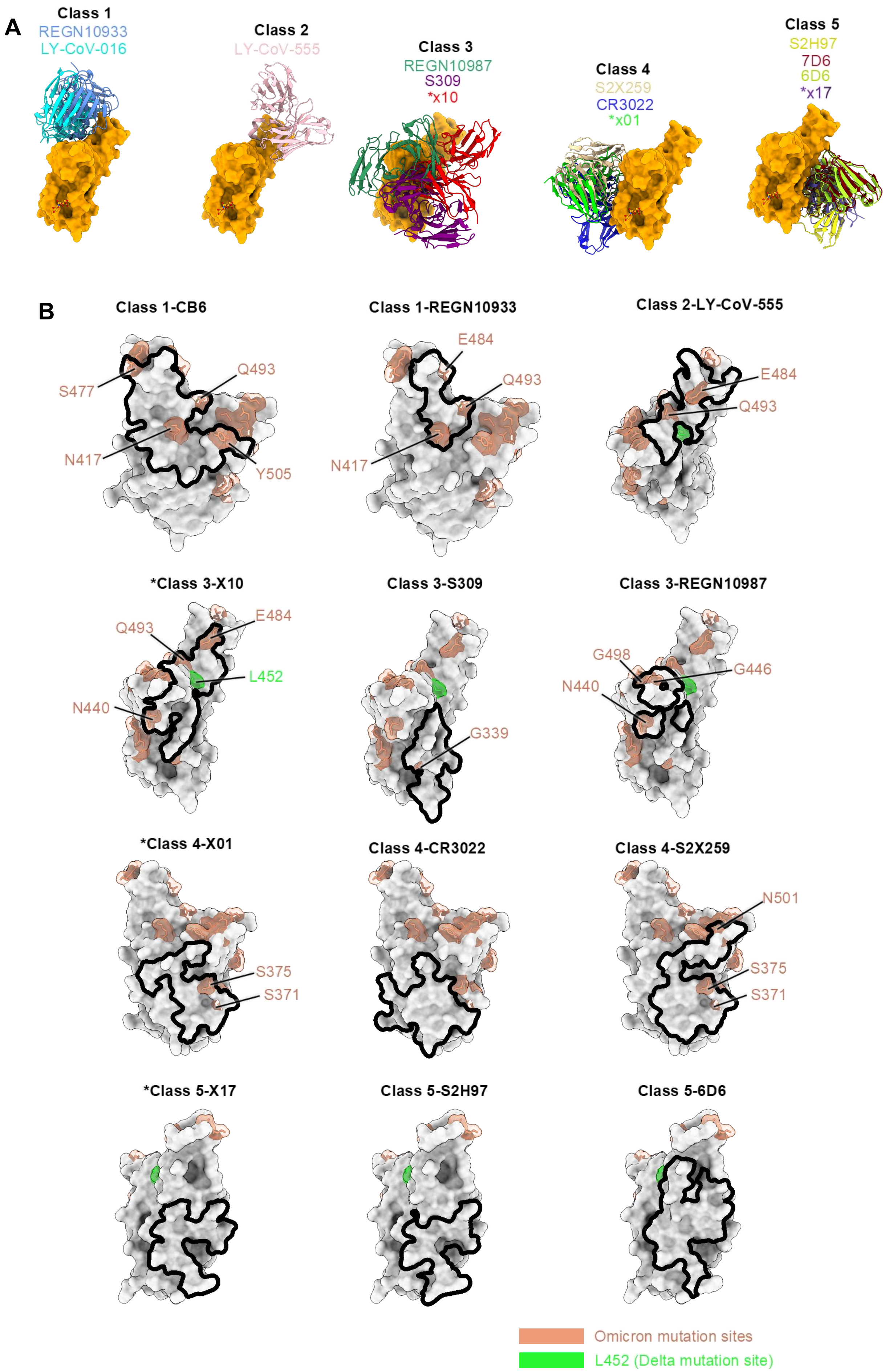
A
Class 1
REGN10933
LY-CoV-016
Class 2
LY-CoV-555
Class 3
REGN10987
S309
*x10
Class 4
S2X259
CR3022
*x01
Class 5
S2H97
7D6
6D6
*x17
B
Class 1-CB6
S477
Q493
N417
Y505
Class 1-REGN10933
E484
Q493
N417
Class 2-LY-CoV-555
E484
Q493
*Class 3-X10
Q493
E484
L452
N440
Class 3-S309
G339
Class 3-REGN10987
G498
G446
N440
*Class 4-X01
S375
S371
Class 4-CR3022
Class 4-S2X259
N501
S375
S371
*Class 5-X17
Class 5-S2H97
Class 5-6D6
Omicron mutation sites
L452 (Delta mutation site)

Fig. S11. Comparisons of the binding modes and epitopes of our three nAbs and other representative nAbs including those authorized in EUA. (A) Comparisons of binding modes between three nAbs (X10, X01 and X17) and other reported nAbs according to the RBD specific antibody-classification (class1-5). X10, X01 and X17 could be classified into Class 3, Class 4 and Class5, respectively. (B) Comparisons of footprints of representative nAbs on WT-RBD (gray surface representation). The 15 Omicron mutation sites are highlighted in coral.

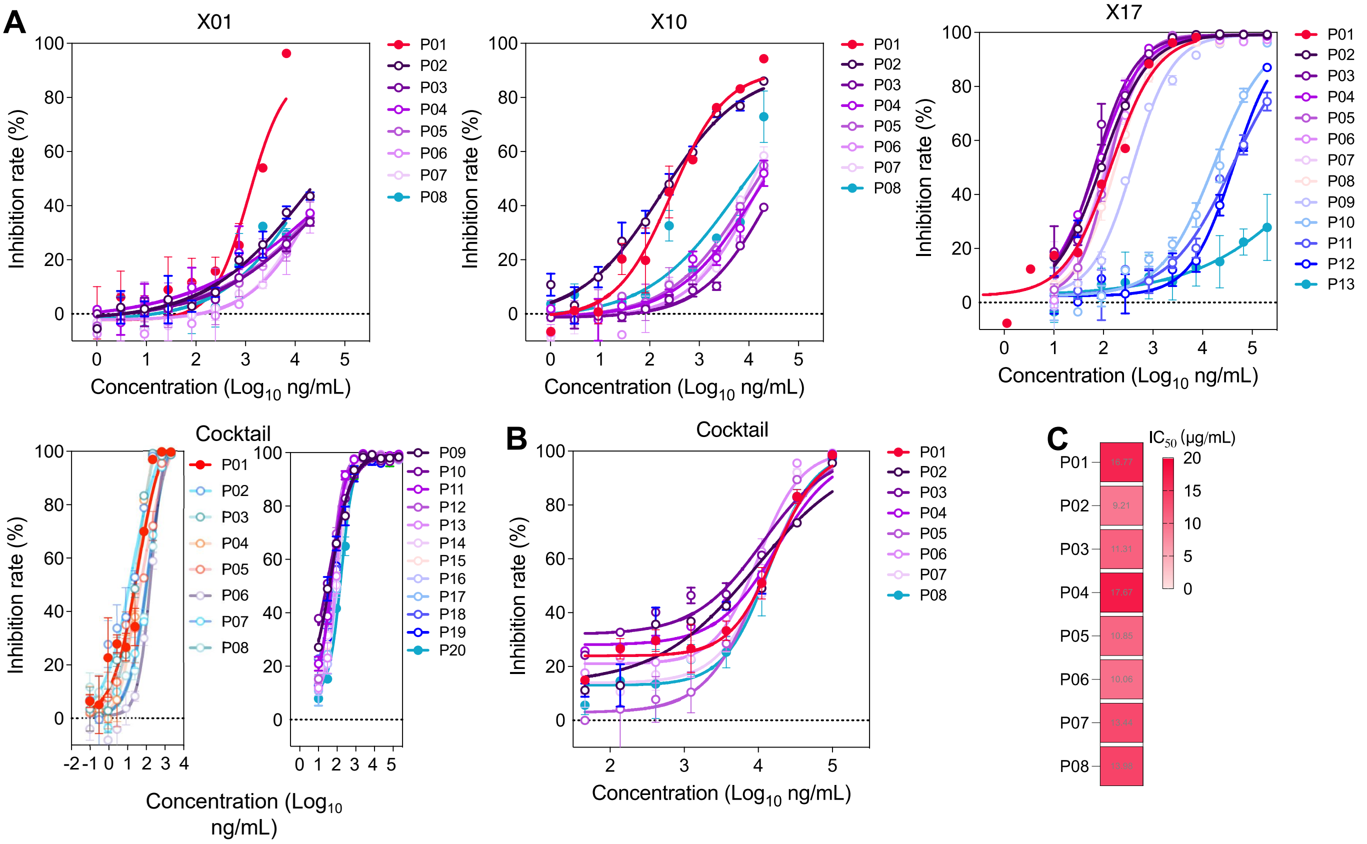

Fig. S12. Viral escape under single antibody and triple-antibody cocktail pressure. (A and B) The curves of single antibodies (X01, X10 and X17) and the triple-antibody cocktail against the replicative rVSV-SARS2 prototype strain (A) and Omicron variant (B) at different passages. (C) IC_{50} values of the triple antibody cocktail against replicative rVSV-SARS2 Omicron variant in different passages. Replicative rVSV-SARS2 Omicron variants in some passages that cause IC_{50} values higher than 20 μg/mL are identified as “complete escape”.

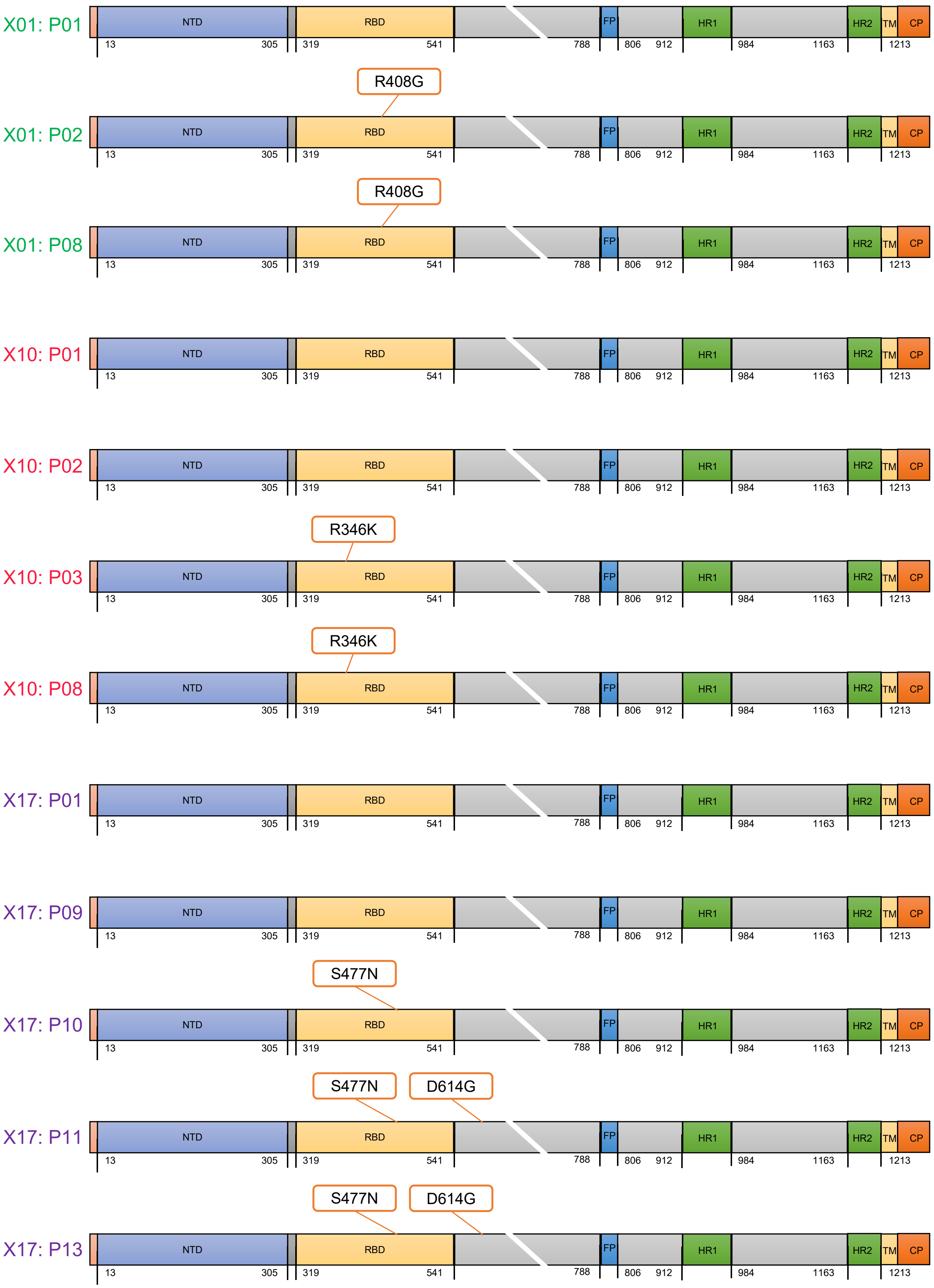

Fig. S13. Amino acid substitutions on the spike of escaped replicative rVSV-SARS2 variants. The spike genes of escaped replicative rVSV-SARS2 variants in the designated passages were sequenced, and amino acid substitutions were marked.

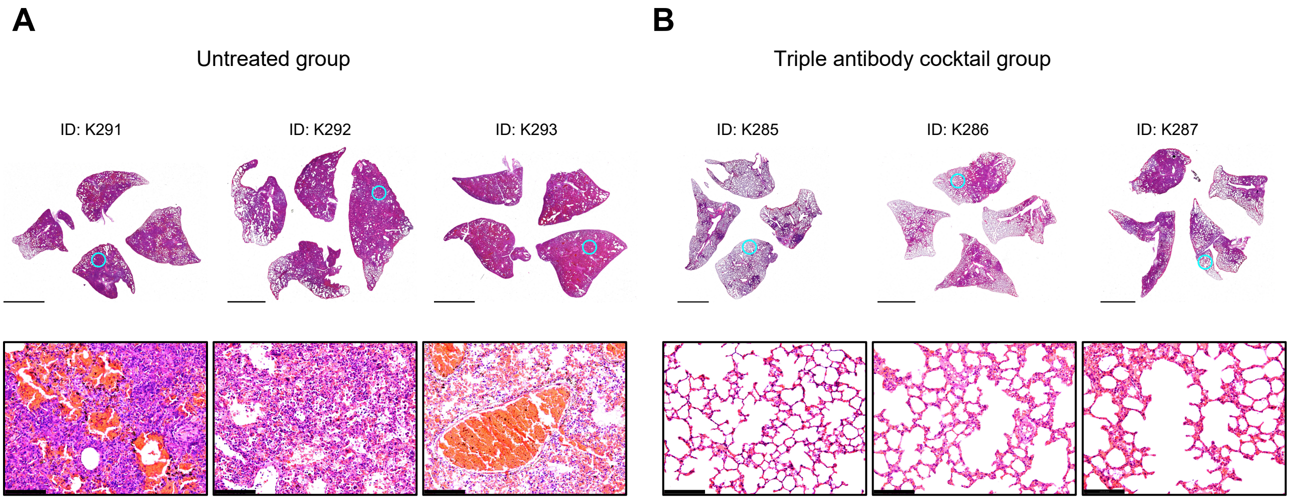

Fig. S14. H&E-staining of four whole lung lobes collected from the PBS (untreated) group (A) and the triple antibody cocktail treated group (B) at 5 dpi. Views of the four lobes are presented in the top panel, and the areas in the cyan circle were enlarged in the bottom panel. Scale bars indicate 4 mm (upper panels) and 124 μm (lower panels).

Table S1. Neutralization potencies of nAbs against SARS-CoV and SARS-CoV-2 prototype strain and VOCs.

ID	class	IC_{50} (ng/mL)															
		SARS-CoV		SARS-CoV-2		D614G		B.1.1.7		B.1.351		B.1.1.28		B.1.617.2		B.1.1.529	
X01	C1	5.3	7.2	30.4	60.5	57.1	115.6	153.4	187.2	53.0	86.6	151.0	111.9	54.1	62.8	38305.0	28203.0
X24	C1	9.1	9.1	27.3	27.5	33.5	29.9	51.1	53.6	26.0	26.4	70.4	86.8	39.8	42.3	984.4	1564.0
X21	C1	47.3	40.7	11.8	11.5	155.9	150.9	174.6	193.3	125.5	100.0	265.4	340.0	30.3	29.8	100000.0	100000.0
X19	C1	25.6	14.5	4.9	9.6	24.0	14.5	6.6	7.9	21.9	14.9	90.6	68.3	33.6	43.2	2592.0	3321.0
X23	C1	5.0	6.5	22.0	26.2	24.1	28.1	44.5	55.2	16.0	18.8	54.2	66.0	35.0	41.2	2053.0	1629.0
X04	C1	18.8	21.5	26.3	39.5	1132.0	1774.0	495.9	409.5	1552.0	3082.0	7320.0	5165.0	719.3	981.4	12177.0	16480.0
X15	C1	47.4	59.4	28.4	36.6	1781.0	6621.0	939.2	862.9	544.1	682.0	2010.0	1542.0	634.3	606.2	27582.0	26294.0
X18	C1	10.8	5.9	68.7	35.1	526.3	477.3	714.1	572.9	113.6	101.6	368.9	310.2	190.1	191.8	100000.0	100000.0
X17	C1	16.5	26.3	62.4	60.6	589.7	719.4	246.7	320.1	246.3	330.6	984.9	1055.0	527.2	560.0	1926.0	1414.0
X10	C1	16.0	15.1	17.2	14.4	35.2	30.7	75.0	66.0	22.7	23.3	89.2	94.6	190.2	153.8	12265.0	12172.0
X34	C1	640.3	375.2	84.5	104.6	48.8	45.4	44.4	40.3	158.6	173.7	151.8	171.3	91.7	85.6	89.8	122.7
X05	C1	20.6	21.1	195.1	210.5	287.9	266.1	487.1	478.0	192.7	164.6	61.9	51.5	149.4	153.1	100000.0	100000.0
X25	C1	32.4	27.4	96.1	94.7	197.4	195.8	319.6	290.3	122.9	140.8	357.6	352.3	245.2	216.4	2223.0	2860.0
X06	C1	135.0	201.3	353.4	602.8	739.9	1000.0	1020.0	1349.0	760.0	783.4	1585.0	1770.0	1206.0	1202.0	9118.0	14212.0
X03	C1	142.5	132.9	209.0	219.3	3339.0	2678.0	3737.0	3408.0	2937.0	3147.0	6970.0	7751.0	2443.0	2347.0	71797.0	77468.0
X02	C1	1882.0	1587.0	188.7	166.7	193.1	213.8	252.2	309.7	153.2	154.1	224.1	268.2	72.6	65.0	100000.0	100000.0
X13	C1	3113.0	2442.0	421.3	438.6	617.4	579.7	836.1	892.4	328.1	306.5	832.9	805.5	808.4	1013.0	100000.0	100000.0
X31	C1	266.8	261.3	528.0	627.0	1707.0	1672.0	1994.0	1910.0	881.1	941.1	1940.0	2067.0	730.0	520.3	2972.0	3175.0
X27	C1	490.0	656.9	2105.0	2570.0	8477.0	10131.0	5199.0	7045.0	5449.0	6146.0	5795.0	6819.0	13005.0	14626.0	21393.0	24152.0
X26	C2	55.5	23.6	932.5	745.4	530.1	279.2	936.5	723.3	401.0	229.1	567.1	590.2	67.9	51.6	100000.0	100000.0
X30	C2	76.2	69.5	1703.0	997.2	1026.0	1422.0	1065.0	1345.0	339.3	588.2	793.1	1064.0	130.8	173.4	1063.0	1098.0
X28	C2	42.0	43.1	1378.0	423.3	1714.0	1656.0	1696.0	1669.0	1050.0	864.1	1102.0	1001.0	291.9	297.7	1151.0	842.4
X29	C2	40.9	33.5	2757.0	3068.0	1096.0	584.7	1048.0	1004.0	525.4	310.4	783.4	548.1	213.5	221.8	678.9	764.5
X20	C3	14473.0	17697.0	0.5	0.8	0.7	0.8	0.9	1.0	0.3	0.4	0.6	0.7	4.9	5.5	100000.0	100000.0
X16	C3	13270.0	12691.0	1.6	1.7	1.6	1.4	1.1	1.2	0.8	0.7	0.9	1.0	7.5	7.4	100000.0	100000.0
X33	C3	14717.0	15188.0	0.5	0.9	0.8	1.3	0.8	1.1	0.1	0.1	0.6	0.4	4.3	3.5	100000.0	100000.0
X12	C3	180.2	196.8	2.9	2.7	3.4	3.2	1.2	1.2	965.3	1078.0	2510.0	2650.0	50.8	44.8	956.1	1684.0
X14	C3	3473.0	1791.0	7.3	4.4	11.3	8.1	3.6	2.2	44.6	18.9	198.5	178.5	30.6	23.0	4061.0	1762.0
X09	C3	9587.0	10181.0	2.2	2.6	4.4	6.0	3.8	4.7	4074.0	3503.0	1780.0	2012.0	20000.0	20000.0	20.9	28.8
X08	C3	8700.0	9083.0	3.3	3.6	5.0	5.1	5.4	6.6	61.5	65.7	139.7	141.2	15.1	16.8	263.6	333.0
X22	C3	4278.0	3097.0	6.2	3.1	7.6	4.1	2.2	1.0	21.1	14.5	77.6	63.1	71.1	51.3	100000.0	100000.0
X32	C3	16120.0	16705.0	4.2	2.4	6.1	2.7	7.2	3.2	20.8	20.7	68.1	48.8	74.3	61.8	13187.0	34347.0
X07	C3	17491.0	16407.0	96.6	86.3	123.1	128.3	137.6	145.0	192.0	182.1	240.3	316.5	30.0	29.8	100000.0	100000.0
X11	C3	6139.0	6927.0	29.3	30.2	25.8	30.7	6910.0	6224.0	3885.0	4007.0	177.2	205.4	1710.0	1479.0	100000.0	100000.0

Table S2. Statistics of Cryo-EM data collection, 3D reconstruction, model refinement and model validation.

	SARS-CoV-2-S:X10:X01:X17	Delta-S:X10:X01:X17	Delta-S:X10:X01:X17-interface	Omicron-S:X10:X01:X17	SARS-CoV-S:X10:X01:X17	SARS-CoV-S:X10:X01:X17-interface
**Data collection and processing**						
Microscope	FEI TF30	FEI TF30	FEI TF30	FEI TF30	FEI TF30	FEI TF30
Camera	K3	K3	K3	K3	K3	K3
Magnification	39,000	39,000	39,000	39,000	39,000	39,000
Voltage (kV)	300	300	300	300	300	300
Electron exposure (e-/A2)	60	60	60	60	60	60
Defocus range (μm)	0.8-2.0	0.8-1.8	1.0-2.0	1.0-2.2	1.0-2.0	0.9-2.3
Pixel size (Å)	0.778	0.778	0.778	0.778	0.778	0.778
Micrographs (total)	4,940	4,240	4,240	1,752	7,148	7,148
Micrographs (used)	4,561	3,526	3,526	1,752	6,471	6,471
Final particle images (no.)	1,028,337	127,022	127,022	10,910	230,838	230,838
Symmetry imposed	C1	C1	C1	C1	C1	C1
Map resolution (Å)	3.48	3.54	3.77	6.56	3.74	3.83
FSC threshold	0.143	0.143	0.143	0.143	0.143	0.143
Map sharpening B factor (Å2)	-161.9	-83	-110.8	-270.7	-109.5	-132.6
**Validation**						
MolProbity score	1.84	/	1.68	/	/	1.69
Clashscore	7.90	/	4.11	/	/	4.70
Poor rotamers (%)	0.14	/	0.27	/	/	0.27
RMS (bonds)	0.0067	/	0.0071	/	/	0.0076
RMS (angles)	1.03	/	1.17	/	/	1.20
Ramachadran plot						
Favored (%)	93.92	/	92.01	/	/	92.90
Allowed (%)	5.96	/	7.87	/	/	4.70
Disallowed (%)	0.12	/	0.12	/	/	0.00
